# mTOR Suppresses Macroautophagy During Striatal Postnatal Development and Is Hyperactive in Mouse Models of Autism Spectrum Disorders

**DOI:** 10.3389/fncel.2020.00070

**Published:** 2020-03-31

**Authors:** Ori J. Lieberman, Veronica Cartocci, Irena Pigulevskiy, Maya Molinari, Josep Carbonell, Miguel Bellés Broseta, Michael R. Post, David Sulzer, Anders Borgkvist, Emanuela Santini

**Affiliations:** ^1^Division of Molecular Therapeutics, Department of Psychiatry, Columbia University Vagelos College of Physicians and Surgeons, New York, NY, United States; ^2^Division of Movement Disorders, Department of Neurology, Columbia University Vagelos College of Physicians and Surgeons, New York, NY, United States; ^3^Department of Neuroscience, Karolinska Institute, Stockholm, Sweden

**Keywords:** striatum, development, autophagy, mTOR, autism, valproic acid

## Abstract

Macroautophagy (hereafter referred to as autophagy) plays a critical role in neuronal function related to development and degeneration. Here, we investigated whether autophagy is developmentally regulated in the striatum, a brain region implicated in neurodevelopmental disease. We demonstrate that autophagic flux is suppressed during striatal postnatal development, reaching adult levels around postnatal day 28 (P28). We also find that mTOR signaling, a key regulator of autophagy, increases during the same developmental period. We further show that mTOR signaling is responsible for suppressing autophagy, via regulation of Beclin-1 and VPS34 activity. Finally, we discover that autophagy is downregulated during late striatal postnatal development (P28) in mice with *in utero* exposure to valproic acid (VPA), an established mouse model of autism spectrum disorder (ASD). VPA-exposed mice also display deficits in striatal neurotransmission and social behavior. Correction of hyperactive mTOR signaling in VPA-exposed mice restores social behavior. These results demonstrate that neurons coopt metabolic signaling cascades to developmentally regulate autophagy and provide additional evidence that mTOR-dependent signaling pathways represent pathogenic signaling cascades in ASD mouse models that are active during specific postnatal windows.

## Introduction

Best characterized in studies of brewer’s yeast, macroautophagy (hereafter referred to as autophagy) is a degradative process for long-lived proteins and damaged organelles (Ohsumi, 2014). In neurons, autophagy is considered to play both protective and pathogenic roles, as it contributes not only to proteostasis and cell survival (Hara et al., 2006; Komatsu et al., 2006; Yamamoto et al., 2006; Yamamoto and Simonsen, 2011), but also to autophagic cell death in neurodegenerative diseases (González-Polo et al., 2005; Wang et al., 2006; Chakrabarti et al., 2009; Yang et al., 2011; Yamamoto and Yue, 2014). Recently, autophagy has been recognized as an important cellular process during neuronal development (Shen and Ganetzky, 2009; Tang et al., 2014; Dragich et al., 2016; Stavoe et al., 2016; Kim et al., 2017; Lieberman et al., 2019a). Autophagic dysfunction is observed in humans with neurodevelopmental disorders (Lee et al., 2013; Poultney et al., 2013; Byrne et al., 2016; Hor and Tang, 2018), and mouse models with reduced autophagy display phenotypes implicated in autism spectrum disorders (ASD) (Tang et al., 2014; Kim et al., 2017; Yan et al., 2018). Importantly, autophagic dysfunction is found in both genetic and environmental animal models of ASD, suggesting its impairment may be a general feature of ASD pathophysiology (Tang et al., 2014; Qin et al., 2016; Zhang et al., 2016, 2019; Wu et al., 2018; Yan et al., 2018). Notably, rodents prenatally exposed to valproic acid (VPA) show a reduction in brain autophagy accompanied by a series of morphological alterations and behavioral impairments consistent with the ASD symptomatology described in patients (Qin et al., 2016; Zhang et al., 2016; Wu et al., 2018; Zhang et al., 2019). Yet, little is known about the developmental regulation of neuronal autophagy and the possible implications for ASD.

Recently, the striatum, which is the main input nucleus of the basal ganglia, a brain circuit controlling action selection and reward processing, has been implicated in the pathophysiology of multiple neurodevelopmental diseases including ASD (Gerfen and Surmeier, 2011; Fuccillo, 2016). Moreover, the principal neurons of the striatum, the spiny projection neurons (SPNs), although they migrate to the striatum during the embryonic period (Song and Harlan, 1994), undergo a significant maturation during the first four postnatal weeks. SPNs receive excitatory inputs from the cortex and thalamus during the second and third postnatal weeks (Tepper et al., 1998). Dopaminergic axons innervate the striatum at birth but their ability to release neurotransmitter increases during the first 2 weeks (Voorn et al., 1988; Lieberman et al., 2018). Finally, the intrinsic excitability of SPNs matures from weeks two through four (Tepper et al., 1998; Peixoto et al., 2016). Notably, autophagy has been proposed to contribute to synaptic maturation and plasticity and dopamine release (Hernandez et al., 2012; Tang et al., 2014; Nikoletopoulou et al., 2017). Thus, establishing whether autophagy is differentially regulated during postnatal development of the striatum and determining whether this temporal regulation is impaired in ASD would provide insights into its role in neurodevelopmental disorders.

Autophagy is a tightly regulated multi-step process (Bento et al., 2016) that, in dividing cells is controlled by energy balance (i.e., nutrient status) and metabolic kinases, including the mammalian target of rapamycin (mTOR). mTOR regulates autophagy via several mechanisms (He and Klionsky, 2009), including by phosphorylating and negatively regulating Unc-51-like autophagy-activating kinase 1 (ULK1) at Ser757 (Jung et al., 2009; Kim et al., 2011). This step prevents ULK1-mediated phosphorylation of Beclin-1 at Ser14, and the subsequent increase of PI3K activity of Vps34 (Russell et al., 2013). These molecular events initiate the formation of preautophagic structures that are subsequently expanded by a molecular cascade resulting in the modification of LC3, one of the mammalian homologs of the yeast Atg8 (Shpilka et al., 2011). Processing of LC3 leads to phagophore expansion and sealing and is used as a biochemical readout of autophagosome formation (Kabeya et al., 2000; Klionsky et al., 2016). The enclosed, mature autophagosome then traffics to the lysosome where the autophagic cargo and cargo adaptors, such as p62, are degraded (Tanida et al., 2005).

Whether similar signaling regulates autophagy in neurons remains controversial. It has been proposed that autophagy may act as a constitutive process for cellular homeostasis, thus circumventing the control of metabolic kinases such as mTOR (Yamamoto and Yue, 2014). The links between nutrient status and autophagy in neurons moreover remain elusive, with reports suggesting a regional and age-specific autophagic response to nutrient deprivation in neurons (Kaushik et al., 2011; Nikoletopoulou et al., 2017) and studies indicating the contrary (Mizushima et al., 2004). Moreover, direct regulation of autophagy by mTOR, independent of nutrient status, has been reported by some (Hernandez et al., 2012; Tang et al., 2014) but not others (Tsvetkov et al., 2010; Maday and Holzbaur, 2016). These contrasting results raise important questions concerning the control of autophagic activity in brain regions implicated in neurodevelopmental diseases, the molecular changes associated with pathologic conditions and the nature of signaling pathways that regulate autophagy in neurons (i.e., signaling operating during nutrient deprivation-induced autophagy in non-neuronal cells).

Using biochemical, pharmacological and histological approaches, we first demonstrate that in SPNs autophagy is dynamically downregulated during postnatal development, following the upregulation of mTOR activity. Then we employ the VPA model of ASD which has construct, face and predictive validity (Chomiak et al., 2013; Roullet et al., 2013; Nicolini and Fahnestock, 2018) to show that in the striatum of ASD model mice, autophagy is specifically reduced during a discrete developmental window (P28). At the same postnatal age, we also observe changes in excitatory synaptic transmission. Finally, we find that mice exposed to VPA *in utero* display a social deficit and that systemic treatment with rapamycin, an inhibitor of mTOR, normalizes it. Our results suggest that autophagy may play temporally-specific roles in brain postnatal development and that it may be one of the pathogenic signaling pathways implicated in neurodevelopmental disorders and ASD.

## Results

### Markers of Autophagic Activity Decrease During Striatal Postnatal Development

To identify changes in autophagic activity during postnatal striatal development, we collected striata from mice at postnatal days 10, 14, 18, 28 and in adults (postnatal day 120; Tepper et al., 1998; Peixoto et al., 2016; Lieberman et al., 2018). These postnatal ages represent critical timepoints for striatal development. Briefly, synaptic dopamine release has begun and interneurons have begun to mature in the striatum at postnatal day 10 (Plotkin et al., 2005; Ferrari et al., 2012; Lieberman et al., 2018). By postnatal day 14, excitatory inputs from the cortex and thalamus arrive and eye opening has occurred, providing higher levels of sensory input. P18 represents the end of synaptogenesis and an age immediately before weaning. At age P28, the period of postnatal refinement has ended (Tepper et al., 1998). We compared tissue from these ages to mice in early adulthood at postnatal day 120.

**FIGURE 1:**
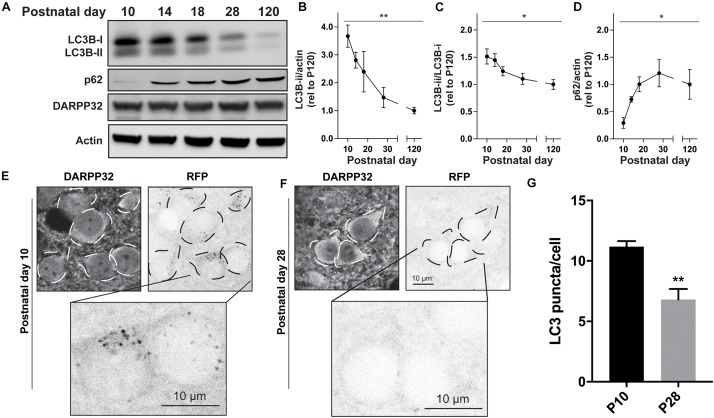
Autophagy decreases during striatal development. **(A)** Representative Western blot images for LC3B, p62, DARPP32, and actin. Quantification of **(B)** LC3B-ii relative to actin, **(C)** LC3B-ii relative to LC3B-i, **(D)** p62 relative to actin normalized to P120 values. Data analyzed with one-way ANOVA; **(B)** Age: *F*_(__4_,_21__)_ = 6.526, *p* = 0.0014; **(C)** Age: *F*_(__4_,_22__)_ = 3.797, *p* = 0.0171; **(D)** Age: *F*_(__4_,_15__)_ = 3.762, *p* = 0.0260. *p < 0.05, **p < 0.01, *n* = 4–6 mice/age. **(E,F)** Representative images of DARPP32 stained striatal neurons and RFP fluorescence from mice aged P10 and P28. Dashed lines indicate cell body outlines. **(G)** Quantification of number of LC3 puncta/cell. Unpaired, two-tailed *t*-test, *t*_4_ = 4.392, *p* = 0.0118. *N* = 3 mice/age, 20–50 cells were analyzed per mouse.

We first measured the levels of DARPP32, a classic SPN marker, and actin as a loading control across postnatal development and found no differences (Figure 1A). We then measured the level of total and processed form of the Atg8 family member, LC3B (Tanida et al., 2005; Klionsky et al., 2016). We found a significant effect of age on the levels of processed LC3B (LC3B-ii) relative to actin and unprocessed LC3B (LC3B-i) (Figures 1A–C). The level of the autophagic adapter protein, p62, whose steady-state levels are determined by its own autophagic degradation, increased over the postnatal period (Figure 1D). These data suggest that overall autophagic activity decreases during the first four postnatal weeks.

### LC3 + Puncta Decrease in Striatal Spiny Projection Neurons During Postnatal Development

Western blot analysis of total striatal lysates includes proteins from all cell types present in the striatum, including neurons, glia, and vascular cells. To define the cell type in which the developmental changes in autophagy occur, we utilized a transgenic mouse ubiquitously expressing LC3 fused to both green (GFP) and red fluorescent proteins (RFPs) (Supplementary Figure S1A; tandem fluorescent-tagged LC3 or tfLC3) (Li et al., 2014). After processing, LC3 transitions from the cytosol to become membrane-bound on the autophagosome (LC3B-ii). Visualizing the distribution of fluorophore-tagged LC3 (LC3 puncta) provides a well-established assay for monitoring autophagic activity within a cell (Klionsky et al., 2016). Furthermore, as the fluorescence of the GFP component is quenched by the low pH of the lysosome, tfLC3 permits analysis of the total number of autophagosomes and autolysosomes (RFP + puncta) and non-acidified autophagosomes (GFP + RFP + puncta).

We first used 2-photon microscopy to simultaneously image GFP and RFP signals in acute brain slices tfLC3 mice at age P10 and P28 (Supplementary Figures S1B,C). At both ages, GFP fluorescence was diffuse in the cytosol and processes of striatal cells and RFP + puncta were present in the soma. The density of the striatal neuropil prevented analysis of tfLC3 + puncta in axons or dendrites (Supplementary Figures S1B,C). Because the level of GFP fluorescence was too strong in the soma to discern individual puncta, we used the number of RFP puncta as a proxy for autophagosomes, with the caveat that we were unable to determine whether these puncta were also fused with lysosomes.

To identify the number of RFP + puncta in specific cell types, we perfused mice at age P10 and P28 and co-labeled with cell-type specific markers of striatal neurons. Spiny projection neurons can be identified by immunostaining with antibodies against DARPP-32. We observed a significant decrease in RFP + puncta in DARPP32 + cells between P10 and P28 (Figures 1E–G), indicating that the reduction in autophagic activity occurs in SPNs. To determine whether autophagy is similarly regulated in other striatal cells, we quantified the number of RFP + puncta in DARPP32- cells. We did not find a significant difference in the number of RPF + puncta in DARRP32- cells between P10 and P28 (P10 = 15 ± 3.14 and P28 = 22.43 ± 4.12; Unpaired, two-tailed *t*-test, ns; *N* = 3 mice/age). These results suggest that the reduction in autophagic activity we measured using biochemical assays of striatal lysates occurs specifically in SPNs.

### Autophagosome Biosynthesis Is Suppressed During Postnatal Development

**FIGURE 2:**
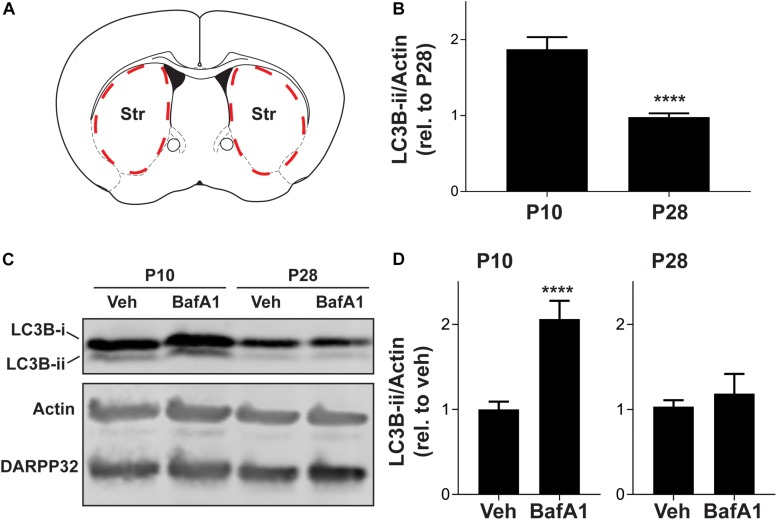
Striatal autophagic flux decreases from P10 to P28. **(A)** Schematic of coronal brain section showing dissection boundaries for *ex vivo* experiments. **(B)** Quantification of LC3B-ii relative to actin for every vehicle-only slice showed in Figures 4, 5. Unpaired, two-tailed *t*-test, *t*_52_ = 5.824, ****p < 0.0001. **(C)** Representative Western blot images of actin, DARPP32 and LC3B in slices obtained from P10 or P28 mice, incubated with BafA1 (100 nM, 3 h) or vehicle (Veh; DMSO, 0.1%). **(D)** LC3B-ii relative to actin, normalized to vehicle condition at each age. P10: unpaired, two-tailed *t*-test, *t*_25_ = 5.113, ****p < 0.0001; P28: unpaired, two-tailed *t*-test, *t*_10_ = 0.6228, *p* = 0.5473. P10: Veh: *n* = 16 slices, BafA1 *n* = 11 slices from 4 to 6 mice. P28: Veh: *n* = 6 slices, BafA1 *n* = 6 slices from three mice.

Changes in the level of LC3B-ii, or the number of LC3 puncta, can arise from increases in autophagosome biosynthesis or decreases in the efficiency of autophagosome maturation and lysosomal degradation. To dissect the changes in autophagy during the postnatal period, we developed an *ex vivo* system to test the effects of drugs that do not cross the blood-brain barrier (Figure 2A). We generated acute brain slices from P10 or P28 mice and removed non-striatal tissue. We found that neither the slice procedure nor the incubation affected the age-dependent reduction in autophagy markers observed *in vivo* (Figures 2A,B compared to Figure 1). This confirms that the mechanisms underlying changes in autophagic markers during postnatal development can be defined using this *ex vivo* system.

We incubated slices from mice at age P10 and P28 with bafilomycin A1 (BafA1, 100 nM), a specific inhibitor of the vacuolar proton pump, or vehicle (DMSO, 0.1%) for 3 h, an incubation time similar to that used in cultured cells (Klionsky et al., 2016), to prevent lysosomal acidification and block autophagosome-lysosome fusion. As treatment with BafA1 prevents LC3B-ii degradation, changes in LC3B-ii levels following BafA1 treatment are interpreted as the rate of autophagosome biosynthesis. BafA1 treatment increased LC3B-ii in slices from P10 mice but, had no significant effect in slices from P28 mice (Figures 2C,D). BafA1 treatment had no effect on DARPP32 levels at either age (Figure 2C). Slices from either age treated with BafA1 for 1 h showed no change in LC3B-ii levels (data not shown). This suggests that the higher baseline level of LC3B-ii at P10 arises from increased autophagosome biosynthesis.

### mTOR Signaling Is Upregulated During the Postnatal Development

mTOR signaling is a key negative regulator of autophagic activity. We therefore hypothesized that mTOR activity in the striatum increases during postnatal development and suppresses autophagy.

**FIGURE 3:**
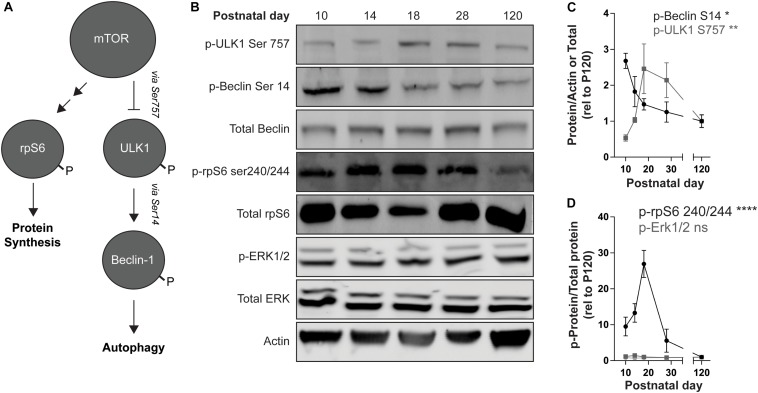
mTOR signaling increases during striatal development. **(A)** Schematic representation of mTOR targets. mTOR inhibits ULK1 activity by phosphorylating Ser757. P-ULK1 activates Vps34 activity (not shown) by phosphorylating Beclin-1 on Ser14. mTOR promotes protein synthesis by activation of S6K-1 (not shown), which phosphorylates rpS6 on Ser240/244. The single and double arrows indicate direct (i.e., ULK1, Beclin-1) and indirect (i.e., rpS6) downstream targets, respectively. **(B)** Representative Western blot images quantified in **(C,D)**. **(C)** Quantification of pULK1 S757 relative to actin (gray squares) and p-Beclin S14 relative to total Beclin-1 (black dots). Data analyzed with one-way ANOVA; p-ULK1 S757/actin: age: *F*_(__4_,_13__)_ = 6.093, *p* = 0.0055; p-Beclin-1S14/total Beclin-1: age: *F*_(__4_,_14__)_ = 4.945, *p* = 0.0107. **(D)** Quantification of p-rpS6 S240/244 and p-Erk1/2 relative to total rpS6 and total Erk1/2, respectively. Data analyzed with one-way ANOVA; p-rpS6 240/244: age: *F*_(__4_,_20__)_ = 12.69, *p* < 0.0001. p-Erk1: age: *F*_(__4_,_17__)_ = 2.625, *p* = 0.0712. p-Erk2: age: *F*_(__4_,_17__)_ = 0.3561, *p* = 0.8362. *N* = 3–6 mice/age. *p < 0.05, **p < 0.01, ****p < 0.0001.

mTOR kinase activity can be monitored by measuring the state of phosphorylation of its downstream targets. The level of phosphorylation at serine 757 of ULK1, which is phosphorylated by mTOR and inhibits ULK1 kinase activity (Kim et al., 2011), increased during the postnatal period in the striatum (Figures 3A–C). We then confirmed that ULK1 kinase activity is inhibited by monitoring the phosphorylation state of Beclin-1, a ULK1 target. Phosphorylation of Beclin-1 at serine 14 decreased during postnatal development (Figures 3A–C). mTOR activity also leads to the indirect phosphorylation of the ribosomal protein S6 (rpS6) on serine 240 and serine 244, via activation of the S6 kinase 1 [S6K1; Figure 3A (Magnuson et al., 2012)]. We observed a sharp increase in rpS6 Ser240/244 at P18 before decreasing into adulthood (Figures 3B,D). Overall these data indicate an increase in mTOR activity during striatal postnatal development. Interestingly, we did not observe a significant effect of age on phosphorylation of ERK1/2 (Figures 3B,D) suggesting that increased mTOR activity during the postnatal period is not associated with a global change in molecular signaling.

### mTOR and vps34 Regulate LC3B-ii Levels During Striatal Postnatal Development

**FIGURE 4:**
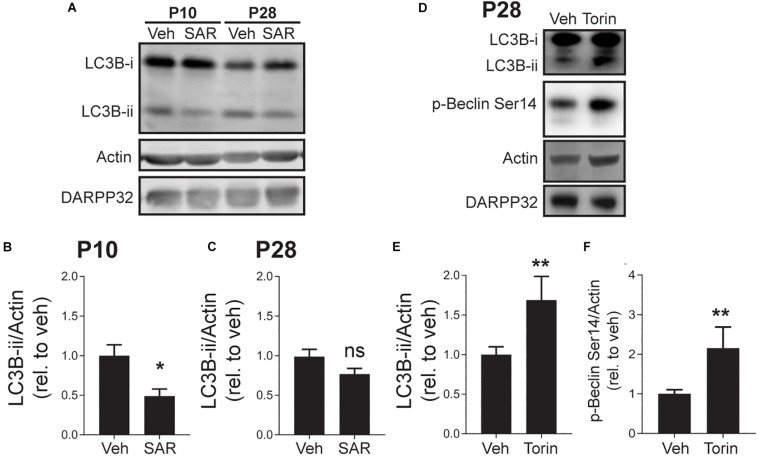
Vps34 activity is required for maintaining LC3B-ii levels at P10 and mTOR inhibition increases p-Beclin-1 and LC3B-ii levels at P28. **(A)** Representative Western blot images for actin, DARPP32 and LC3B-i and -ii in striatal slices obtained from P10 or P28 mice, treated with SAR405 (1 μM) or vehicle (Veh; DMSO, 0.1%). **(B,C)** Quantification of LC3B-ii relative to actin, normalized to vehicle condition at each age. **(B)** P10: unpaired, two-tailed *t*-test, *t*_11_ = 2.985, *p* = 0.0124; P28: unpaired, two-tailed *t*-test, *t*_24_ = 1.807, *p* = 0.0922. P10: Veh: *n* = 6 slices, SAR405 *n* = 7 slices from 3 to 4 mice/age. **(C)** P28: Veh: *n* = 9 slices, Baf *n* = 7 slices from 3 to 4 mice/age. *p < 0.05. **(D)** Representative Western blot images for actin, DARPP32, p-Beclin-1 Ser14 and LC3B-i and -ii in striatal slices from P28 mice, treated with Torin-1 (5 μM) or vehicle (Veh; DMSO, 0.1%). Quantification of **(E)** LC3B-ii and **(F)** p-Beclin S14 relative to actin, normalized to vehicle. Data analyzed with unpaired, two-tailed *t*-test; **(E)** LC3B-ii/actin, *t*_24_ = 2.853, *p* = 0.0088. Veh: *n* = 19 slices, Baf *n* = 7 slices from 5 to 7 mice. *p < 0.05. **(F)** p-Beclin S14/actin, *t*_22_ = 3.337, *p* = 0.0030. **p < 0.01, Veh: *n* = 18 slices, Torin-1 *n* = 6 slices from 3 to 5 mice.

mTOR negatively regulates autophagosome biosynthesis by inhibiting ULK1 activity (Jung et al., 2009; Kim et al., 2011). When ULK1 is active, it promotes autophagosome formation by phosphorylating Beclin-1, which increases the PI3K activity of its partner, Vps34 (Russell et al., 2013). To address whether elevated autophagy at P10 was a result of enhanced Vps34 activity, we incubated acute striatal slices with the Vps34 inhibitor, SAR405 (1 μM) or vehicle (DMSO, 0.1%) for 3 h (Ronan et al., 2014). SAR405 significantly reduced the level of LC3B-ii in slices from mice at age P10 but had no effect on slices from P28 mice (Figures 4A–C). This demonstrates that elevated autophagic activity in the striatum of early postnatal mice is Vps34-dependent. The lack of effect of SAR405 on autophagic activity at P28 indicates that reduced Vps34 activity, possibly via increased mTOR signaling (Figure 3), is responsible for the lower levels of autophagic flux at P28. As predicted SAR405 does not change the levels of phosphorylated Beclin-1 at P10 (veh: 100 ± 9.433 and SAR405: 103.2 ± 6.982; Unpaired, two-tailed *t*-test, ns. *n* = 4 slices/3 mice) and P28 (veh: 100 ± 11.89 and SAR405: 85.29 ± 6.813; Unpaired, two-tailed *t*-test, ns. *n* = 5 slices/3 mice), indicating that there are no regulatory loops altering the activation of Beclin-1 in response to inhibition of Vsp34 activity.

Having shown that Vps34 drives elevated autophagy at P10, we explored whether enhanced mTOR activity inhibits autophagy at P28. mTOR activity can be pharmacologically inhibited by direct active site inhibitors, such as Torin-1 (Thoreen et al., 2009). We incubated striatal slices from mice at P28 with Torin-1 (5 μM) or vehicle (DMSO, 0.1%) for 3 h and measured LC3B-ii levels. Torin-1 treatment increased LC3B-ii levels and the phosphorylation of Beclin-1 at the ULK1 site (Ser14), suggesting that mTOR inhibition activates autophagy in a ULK1/Beclin-1 dependent manner (Figures 4D–F).

### Prenatal VPA Exposure Determine a Decrease in Autophagy During the Late Postnatal Period

To gain insights into the involvement of autophagy in neurodevelopmental disorders and ASD, we asked whether autophagic markers are dysregulated during the postnatal striatal development in the VPA mouse model of ASD.

**FIGURE 5:**
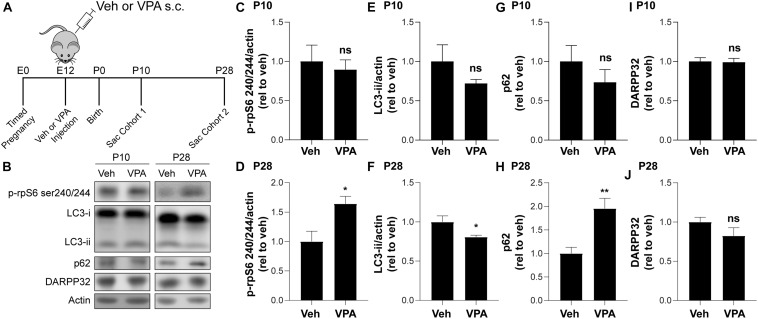
VPA reduces autophagy at P28. **(A)** Schematic of VPA administration. **(B)** Representative Western blot images for LC3B, p62, p-rpS6 S240/244, DARPP32, and actin. **(B–G)** Quantification of **(B–D)** p-rpS6 S240/244 relative to actin, **(B,E,F)** LC3B-ii relative to actin **(B,G,H)** p62 and **(B,I,J)** DARPP32 normalized to VPA untreated (veh, vehicle) mice at P10 and P28, respectively. Data analyzed with unpaired, two-tailed *t*-test; **(C)** p-rpS6 S240/244/actin at P10, ns; **(D)** p-rpS6 S240/244/actin at P28, *t*_11_ = 2.882, *p* = 0.0149 **(E)** LC3B-ii/actin at P10, ns; **(F)** LC3B-ii/actin at P28, *t*_10_ = 2.231, *p* = 0.047; **(G)** p62 at P10, ns; **(H)** p62 at P28, *t*_10_ = 3.752, *p* = 0.0038; **(I)** DARPP32 at P10, ns, and **(J)** DARPP32 at P10, ns, *p < 0.05, **p < 0.01, ns not significant, *n* = 4–7 mice/treatment.

We examined the effects of prenatal VPA exposure [600 mg/kg administered subcutaneously to pregnant mothers at gestational day 12 (Roullet et al., 2013)] on autophagy by collecting the striata of vehicle (Veh) or VPA-treated (VPA) mice at P10 and P28 (Figure 5A). Interestingly, we found a significant elevation in phosphorylation of rpS6 at Ser240/244 at P28 but not at P10 in VPA-treated mice compared to vehicle (Figures 5B–D), suggesting that mTOR signaling was increased following *in utero* VPA exposure. In agreement, we observed a significant decrease in LC3B-ii and an increase of p62 at P28 but not at P10 in VPA-treated mice compared to vehicle (Figures 5B,E–H). We verified that we enriched for striatal tissue in all groups by probing for DARPP-32 (Figures 5B,I,J). Overall, these data suggest that prenatal VPA exposure affects mTOR signaling and autophagy only during the late striatal postnatal development (P28).

### Prenatal VPA Exposure Induces Impairments in SPNs Synaptic Transmission

Because autophagy is important for synaptic function (Hernandez et al., 2012; Tang et al., 2014; Nikoletopoulou et al., 2017) and prenatal VPA exposure affects synaptic transmission in the hippocampus and cortex (Roullet et al., 2013), we determined whether the reduction in autophagy induced by VPA at P28 was also accompanied by a functional change in striatal synaptic transmission.

**FIGURE 6:**
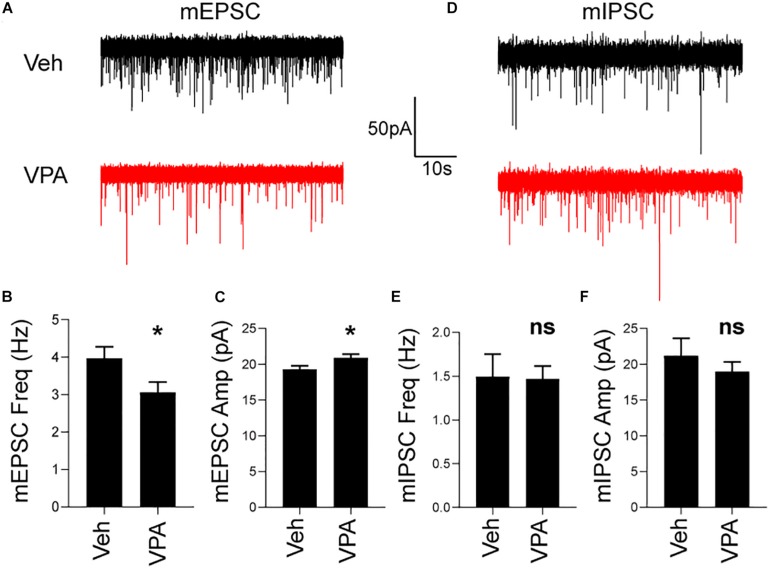
*In utero* VPA exposure impairs striatal excitatory synaptic transmission at P28. **(A)** Representative voltage-clamp recordings of mEPSCs from SPNs in Veh or VPA treated mice. **(B)** Reduction in mEPSC frequency in VPA-treated mice. Veh: *N* = 19 cells, VPA: *N* = 21 cells. *t*_38_ = 2.246, *p* = 0.0306. **(C)** Increased in mEPSC amplitude in VPA-treated mice. Veh: *N* = 19 cells, VPA: *N* = 21 cells. *t*_38_ = 2.113, *p* = 0.0412. **(D)** Representative voltage-clamp recordings of mIPSCs from SPNs in Veh or VPA-treated mice. No difference in mIPSC **(E)** frequency or **(F)** amplitude. Veh: *N* = 10 cells, VPA: *N* = 19 cells. *p < 0.05, ns not significant.

To this end we prepared acute brain slices from P28 veh- and VPA-treated mice and recorded excitatory and inhibitory synaptic transmission in SPNs. In VPA-treated mice, we discovered a decrease in the frequency and increase in the amplitude of mEPSCs compared to vehicle treated mice (Figures 6A–C). In contrast, prenatal exposure to VPA did not affect mIPSC frequency or amplitude at P28 (Figures 6D–F). This data suggest that impaired excitatory transmission occurs in SPNs of mice prenatally exposed to VPA.

### Prenatal VPA Exposure Induces Alteration in Social Behavior That Is Normalized by Treatment With Rapamycin

Since repetitive behaviors and social impairments are required for ASD diagnosis [Diagnostic and Statistical Manual of Mental Disorders [DSM-5^®^], 2013] and are present in genetic models of ASD (Kwon et al., 2006; Ehninger et al., 2008; Zhou et al., 2009; Tsai et al., 2012, 2018; Gkogkas et al., 2013; Santini et al., 2013; Huynh et al., 2015) and VPA-treated rats (Cartocci et al., 2018), we asked whether prenatal exposure to VPA could result in altered repetitive and social behaviors.

We utilized the marble burying test to determine the presence of repetitive behaviors in VPA-exposed or control mice (Thomas et al., 2009, 2012; Henderson et al., 2012; Silverman et al., 2012; Huynh et al., 2014). In this test, the mice are free to explore a cage with 20 marbles arranged in a grid on the surface of the bedding. After 20 min of exploration, we counted the marbles that were buried by the mice. We did not find a significant difference in the number of marbles buried by control and VPA-exposed mice (vehicle-treated mice = 12.86 ± 1.1, VPA-treated mice = 12.57 ± 1.288. Unpaired, two-tailed *t*-test, ns. *N* = 7 mice/treatment). This result suggests that the onset of the repetitive behaviors occurs later than P28 for VPA-exposed mice.

**FIGURE 7:**
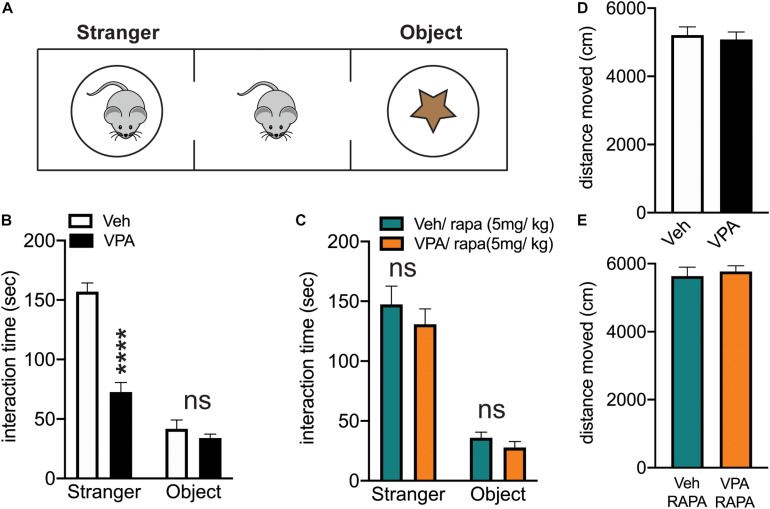
*In utero* exposure to VPA reduces social approach behavior which is rescued by mTOR inhibition**. (A)** Schematic of three chamber arena. Subjects are placed in the center chamber and allowed to explore for 10 min. After this, a conspecific mouse (stranger) is placed in one side chamber and an object is placed in the other side chamber. The subject is allowed to explore the entire arena for 10 min and time spent interacting with either the conspecific (stranger) or the object is recorded. **(B)** The interaction time with stranger and object for control (Veh) and VPA-exposed mice (VPA). Two-way repeated measures ANOVA: treatment x chamber *F*_(__1_,_12__)_ = 30.48, ****p < 0.0001. **(C)** Rapamycin treated Veh and VPA exposed mice show no difference in interaction time with the stranger. Two-way repeated measures ANOVA: treatment x chamber *F*_(__1_,_12__)_ = 0.1397, *p* = 0.7151. Treatment: *F*_(__1_,_12__)_ = 1.584, *p* = 0.2321. Chamber: *F*_(__1_,_12__)_ = 89.93, *p* < 0.0001. **(D,E)** No difference in locomotor activity between Veh and VPA-exposed mice at baseline or following rapamycin treatment. **(D)** unpaired, two-tailed *t*-test, *t*_12_ = 0.3940, *p* = 0.7005. **(E)** Unpaired, two-tailed *t*-test, *t*_12_ = 0.4253, *p* = 0.6781.

We utilized the three chamber social arena to assess social behavior in VPA-exposed or control mice (Moy et al., 2008). After a period of habituation (10 min) to the arena, the mice are free to interact for another 10 min with another conspecific mouse (stranger) or with an object confined inside wire cups in the right and left chambers of the arena (Figure 7A). We found that VPA exposed mice spent significantly less time interacting with the social cue as compared to vehicle exposed mice (Figure 7B).

Since we found that VPA-exposed mice displayed an impairment in social behavior, We also investigated whether inhibiting mTOR activity with rapamycin would result in a normalization of the social abnormalities as described in genetic models of ASD (Zhou et al., 2009; Tsai et al., 2012, 2018; Tang et al., 2014). Rapamycin crosses the blood brain barrier, it has been extensively used *in vivo* to inhibit the activity of mTOR in the multiprotein complex 1 (mTORC1) and, rescue ASD-like behaviors (Zhou et al., 2009; Tsai et al., 2012, 2018; Tang et al., 2014). We treated VPA-exposed and control mice with rapamycin (5 mg/kg; injected intraperitoneally (i.p.) once a day) for 4 days, a treatment that was previously showed to be effective in blocking mTOR in the brain (Ehninger et al., 2008; Santini et al., 2009; Huynh et al., 2014). On the fifth day, 1 h after the rapamycin injection, we subjected the mice to the three chamber arena test. Rapamycin normalized the behavior of VPA-exposed mice. Thus, after rapamycin treatment, the interaction time displayed by vehicle and VPA-treated mice is similar (Figure 7C).

Finally, one possible explanation for the social behavior displayed by VPA-exposed mice (at baseline and following rapamycin treatment) is a change in total locomotor activity. We quantified the total locomotor activity of the mice in the three chamber arena and we found no difference between groups (Figures 7D,E), suggesting that the changes in the interaction time with the social cue are not caused by alteration in exploratory behavior.

## Discussion

Neuronal autophagy has been proposed to play a key role in neurodevelopment and autophagic dysfunction may lead to neurodevelopmental disorders and ASD (Tang et al., 2014; Dragich et al., 2016; Kim et al., 2017; Yan et al., 2018; Lieberman et al., 2019b). In this study we address whether autophagic activity is developmentally controlled in the principal neurons of the striatum, a brain region implicated in neurodevelopmental disorders (Fuccillo, 2016). We found that autophagy is dynamically regulated in SPNs during postnatal development, reaching adult levels around P28. These findings provide mechanistic insight into the regulation of autophagy during striatal postnatal development and establish the basis for a further evaluation of its role in physiological and pathological conditions.

The involvement of the striatum in ASD pathophysiology have been suggested by clinical and experimental observations (Fuccillo, 2016). Magnetic resonance imaging (MRI) and functional-MRI studies indicate anatomical and task-specific alterations in the striatum of ASD patients (Sears et al., 1999; Langen et al., 2007, 2009; Shafritz et al., 2008; Delmonte et al., 2012). Moreover, environmental and genetic animal models of ASD have contributed to conclusively demonstrate a causal relationship between striatal functions and pathophysiological alterations ASD-like (Arndt et al., 2005; Centonze et al., 2008; Shafritz et al., 2008; Shmelkov et al., 2010; Peça et al., 2011; Peñagarikano et al., 2011; Dölen et al., 2013; Santini et al., 2013). Interestingly, we have recently discovered that autophagy is differentially involved in the control of the synaptic functions and excitability of the two subtypes of SPNs (i.e., direct and indirect SPNs; Lieberman et al., 2020). Moreover, mice with specific genetic deletion of atg7, a protein required for autophagy, either in direct or indirect SPNs display hyperactivity, stereotypes and behavioral abnormalities consistent with ASD (Lieberman et al., 2020). Given the dynamic regulations of autophagy occurring in the SPNs during postnatal development and the ASD-like phenotypes displayed by mice with genetic ablation of autophagy in SPNs, the regulation of autophagy during early postnatal development of SPNs may be one of the cellular process disrupted in ASD.

By analyzing key endogenous biochemical markers (such as LC3B-ii, p62, p-Ser757-ULK1, and p-Ser14-Beclin-1), we discovered that autophagy decreases progressively during the postnatal development of SPNs. The autophagy markers were measured in bulk lysates containing neurons, glia and endothelial cells (among others). To identify the cell types that feature changes during postnatal development, we utilized tfLC3 mice (Li et al., 2014). Given the challenges in assaying endogenous autophagic proteins using immunofluorescence, tfLC3 mice provide a unique resource, as overexpressed LC3 is fused to fluorescent proteins that permit morphological characterization of autophagic structure. While the high levels of expression prevented the identification of individual autophagic puncta in both live imaging (Supplementary Figure S1) and fixed tissue (not shown), the level of autophagolysosomes represented by RFP-LC3 + puncta was higher in SPN somata at P10 than at P28, in agreement with our biochemical analysis (Figure 1).

Autophagic flux is determined by the kinetics of autophagosome biosynthesis, autophagosome maturation and autophagic cargo degradation by lysosomal proteases. Steady-state measurement of LC3B-ii levels provide a useful proxy for the measurement of autophagic activity. An elevated level of LC3B-ii could indicate either increased or decreased autophagic flux, as both increased autophagosome biosynthesis *and* decreased lysosomal degradation of autophagic proteins can increase LC3B-ii (Kabeya et al., 2000; Klionsky et al., 2016). To functionally measure autophagic flux in striatal tissue, we developed an *ex vivo* acute brain slice system and found that steady state increases in LC3B-ii levels at P10 were further elevated by acute blockade of lysosomal degradation with BafA1 compared to LC3B-ii levels at P28. This suggests that increased autophagic flux is responsible for the higher steady-state LC3B-ii levels, as inhibition of lysosomal degradation would not have an effect on LC3B-ii levels if lysosomal degradation were already compromised. These results indicate that the postnatal suppression of autophagy results from changes in autophagosome biosynthesis or maturation.

In line with changes in autophagosome biosynthesis/maturation during striatal development, we found that mTOR-dependent signaling increases over the course of postnatal development, thereby suppressing autophagosome synthesis via inhibition of ULK1 and Beclin-1 activity. These include downstream targets involved in protein synthesis, such as rpS6, and autophagy, such as ULK1. In this regard, it is interesting to note that the phosphorylation state of rpS6 follows a slightly different pattern than ULK-1 during postnatal development. One of the reasons of this discrepancy may be the different phosphorylation kinetic of activated mTOR on the rpS6, which is an indirect target regulated via S6K-1 (Magnuson et al., 2012), and on ULK-1, which is a direct target (Kim et al., 2011). Alternatively, a reduction in phosphorylated rpS6 may be the result of the enhanced activity of protein phosphatase 1 (PP1), which dephosphorylates rpS6 (Belandia et al., 1994). It has been showed that the phosphorylation of rpS6 in SPNs is determined by the suppression of dephosphorylation via PKA/DARPP-32-mediated inhibition of PP1 (Bonito-Oliva et al., 2013). Thus, it is possible that a reduction in PKA/DARPP-32 results in an increased PP1 activity which, in turn dephosphorylates rpS6. Finally, a reduction of phosphorylated rpS6 may also be achieved by increased activity of PP2A-like phosphatases, which dephosphorylate and reduce the activation of S6K-1 (Magnuson et al., 2012).

mTOR specifically phosphorylates ULK1 at Ser757 and inhibits its kinase activity (Kim et al., 2011). Consistently, we also observed a decrease in the level of phosphorylation on the ULK1 site in Beclin-1, a key component of the class III-PI3K complex required for autophagosome biosynthesis (Itakura et al., 2008; Russell et al., 2013). These results provide a functional readout for increased mTOR activity on autophagy-specific downstream targets and demonstrate a direct link between mTOR signaling and a regulation of autophagy activity observed during the postnatal development. Inhibition of mTOR activity at P28, when mTOR activity is elevated, induces autophagy and increases the phosphorylation of Beclin-1 at Ser14 and LC3B-ii levels. Further work will also focus on whether additional kinases which integrate metabolic status in the periphery, such as AMPK (He and Klionsky, 2009), control developmental changes in neuronal autophagy.

These findings may help to resolve conflicting results present in the literature. Several groups have found that mTOR inhibition fails to increase autophagic activity in primary neuronal culture (Tsvetkov et al., 2010; Maday and Holzbaur, 2016) or *in vivo* (Fox et al., 2010). In contrast, others report that pharmacological (Hernandez et al., 2012; Tang et al., 2014) or genetic inhibition of mTOR drives autophagy (Yan et al., 2018) as well as hyperactivation of mTOR signaling inhibits autophagy in the CNS (McMahon et al., 2012; Tang et al., 2014; Ebrahimi-Fakhari et al., 2016). This discrepancy may arise from model system used (i.e., cell culture vs. *in vivo*), developmental stage, or treatment paradigm. Defining the substrates that mTOR acts on during brain development is critically important given recent approvals of mTOR inhibitors in neurodevelopmental disease (French et al., 2016; Curatolo et al., 2018). Our findings suggest that mTOR signaling is important for the regulation of autophagy during critical developmental windows in neurons in a manner unrelated to nutrient status. mTOR signaling is under the control of several neural-specific signals such as patterned neuronal activity and specific neurotransmitters or neuromodulators (Yin et al., 2006; Santini et al., 2009; Auerbach et al., 2011; Bockaert and Marin, 2015; Sutton and Caron, 2015), suggesting possible mechanisms through which mTOR activity is regulated within developing neural circuits. There is a broad range of significant changes in neurotransmitter content, neuronal firing patterns and synaptic plasticity that occurs during this period, providing a plethora of candidates that could dynamically regulate mTOR signaling and autophagy during the course of postnatal development. Autophagy is also known to regulate surface levels of neurotransmitter receptors, axon pathfinding, synaptic maturation and plasticity, suggesting that autophagy acts dynamically during early postnatal developmental to entrain adult-like neuronal activity that subsequently suppresses autophagy by activating mTOR signaling (Rowland et al., 2006; Hernandez et al., 2012; Shehata et al., 2012; Tang et al., 2014; Dragich et al., 2016; Nikoletopoulou et al., 2017). Such a feedback process may provide a temporal and mechanistic framework for future studies that address the role of autophagy, and its regulation by mTOR, in neuronal development.

The VPA animal model of ASD, a non-genetic model extensively used to study the neurobiology of ASD and to test for novel behavioral and pharmacological treatments thank to its face, construct and predictive validity (Chomiak et al., 2013; Roullet et al., 2013; Nicolini and Fahnestock, 2018). Prenatal exposure to VPA in humans is associated with neural tube defects and other congenital malformations (Jentink et al., 2010; Werler et al., 2011), neurocognitive impairments, including low IQ (Meador et al., 2013) and high risk of ASD (Bromley et al., 2013; Christensen et al., 2013). Similarly, in rodents, VPA induces a robust range of ASD-like behavioral and morphological alterations (reviewed in Chomiak et al., 2013; Roullet et al., 2013; Nicolini and Fahnestock, 2018). Adult rodents exposed to VPA display a reduction of brain autophagy in associations with the behavioral and morphological alterations (Qin et al., 2016; Zhang et al., 2016, 2019; Wu et al., 2018). In line with these results, we found altered autophagic markers, such as decreased LC3B-ii and increased p62, in the striatum of P28 mice prenatally exposed to VPA. Moreover, we discover that at P10 the VPA does not significantly change autophagy. Thus, it seems that prenatal exposure to VPA determines a detectable reduction of striatal autophagy only during the late postnatal development (P28) that it is maintained in the adulthood (Qin et al., 2016; Zhang et al., 2016, 2019; Wu et al., 2018). The lack of detectable change in autophagy at P10 may be the result of the high autophagic flux that masks the impairment in autophagy. Alternatively, it may be due to an ongoing accumulation of autophagic markers that are under detectable threshold at those early time points.

Consistent with our data demonstrating a direct link between dynamic change in mTOR activity and autophagy during postnatal development of SPNs, we found an increased phosphorylation of rpS6 in VPA treated animals at P28. This result suggests that autophagy and protein synthesis are concomitantly dysregulated through aberrant mTOR activity.

Impaired protein synthesis and autophagy have also been described in genetic ASD animal models (Bagni and Zukin, 2019) and the presence of similar changes also in the non-genetic VPA model may indicate that these represent pathogenic signaling pathways underlying ASD. This molecular characterization may be of importance for early detection and therapeutic treatment of ASD.

We also discovered that reduction of autophagy in VPA-treated mice is accompanied by changes in synaptic transmission at P28. We found a decrease in frequency and increase in amplitude of mEPSCs, which may indicate a reduction in the number of spines and an increase in the strength of the synaptic connection in the striatum of mice prenatally exposed to VPA. This synaptic phenotype is in line with a previous publication reporting a reduction of corticostriatal synapses in the rostral striatum of VPA treated mice (Kuo and Liu, 2017). Moreover, decreased mEPSCs frequency in VPA treated mice is also present in the cerebellar cortex (Wang et al., 2018). Reduction in excitatory striatal inputs is also observed in genetic mouse models of ASD (Fuccillo, 2016).

Finally, we found that mice exposed prenatally to VPA display altered social interaction in the three chamber arena test. This is similar to what is observed in rats treated with VPA (Cartocci et al., 2018). Systemic treatment with rapamycin (5 mg/kg/day, injected i.p. for 5 days) normalizes the social impairments of VPA treated mice. We are aware of the fact that rapamycin will likely affect all the molecular pathways regulated by mTOR, including protein synthesis which is an important signaling cascade involved in ASD (Huynh et al., 2014). Moreover, the systemic administration of rapamycin leads to an inhibition of mTOR not only in the striatum but also in other brain regions regulating social behaviors. Further studies with more specific autophagy inhibitors administered locally or with genetic manipulation of autophagic regulators restricted to the striatum are needed to conclusively demonstrate the involvement of striatal autophagy in the generation of social deficits in the VPA animal model.

In summary, we find that the postnatal development of SPNs is correlated with a change in autophagy and this is determined by mTOR signaling status. We also find that in ASD model mice, mTOR-dependent autophagy is downregulated in SPNs and it is accompanied by impairments in synaptic transmission and social behavior in the late postnatal development.

## Materials and Methods

### Animals

Breeder pairs of C57/BL6J were obtained from Jackson Laboratories (Bar Harbor, ME, United States). Mice were checked every day or every other day for pregnancy and new litters. TfLC3 mice were obtained from Jackson Laboratories [C57BL/6-Tg(CAG-RFP/EGFP/Map1lc3b)1Hill/J; Strain No. 027139]. Breeder pairs were housed on a 12-h light/dark cycle with water and food available *ad libitum*. Offspring were weaned between postnatal day 18 and 20 and split into same sex groups of 2–5. Mice of both sexes were utilized for experiments in Figures 1–4 and data were combined as no effect of gender was observed.

For the VPA experiments, pregnant female breeders received a single subcutaneous injection of VPA at the dose of 600 mg/kg during gestational day 12 (Lauber et al., 2016; Kuo and Liu, 2017; Wang et al., 2018). Control pregnant females received an equivalent dose of vehicle at gestational day 12. Only male mice of the offspring were utilized for these experiments (in Figures 5–7) (Lauber et al., 2016; Kuo and Liu, 2017; Wang et al., 2018).

All experimental procedures were approved by the Columbia University Institutional Animal Care and Use Committee and was conducted in accordance with animal care guidelines of National Institute of Health.

### *In vivo* Sample Preparation

Striatal sample preparation was performed as previously described (Santini et al., 2007). Mice at specified ages were rapidly decapitated and their head was briefly placed in liquid nitrogen. The brain was subsequently removed and a single striatum was dissected and flash frozen in liquid nitrogen. Samples were stored at −80°C until the full cohort was collected. Samples were then homogenized in 1% SDS by a brief sonication. Protein content was determined by the BCA assay (Thermo Fisher). No significant effect of age was found on total protein (data not shown). Samples were then boiled in sample buffer and frozen until western blot analysis.

### Acute Brain Slice

Acute brain slices were prepared essentially as described (Lieberman et al., 2018). Briefly, mice underwent cervical dislocation and the brain was removed and placed in ice-cold high sucrose cutting solution (in mM): 10 NaCl, 2.5 KCl, 25 NaHCO_3_, 0.5 CaCl_2_, 7 MgCl_2_, 1.25 NaH_2_PO_4_, 180 sucrose, 10 glucose bubbled with 95% O_2_/5% CO_2_ to pH 7.4. Brains were mounted on a VT1200 vibratome (Leica Biosystems) and coronal sections (250 μm) including the striatum were collected. For the biochemistry experiments, slices were then transferred to a basin contained ice-cold cutting solution and the striatum was manually dissected. Slices were moved to scintillation vials containing 7 mL of ACSF (in mM): 125 NaCl, 2.5 KCl, 25 NaHCO_3_, 2 CaCl_2_, 1 MgCl_2_, 1.25 NaH_2_PO_4_, and 10 glucose bubbled with 95% O_2_/5% CO_2_ to pH 7.4 at 34°C. Slices from two mice at the same age were combined in individual experiments and split into four conditions. Slices were allowed to rest for 1 h followed by addition of vehicle or drug. Following incubation with drug at 34°C for the specified time, slices were removed and flash frozen in liquid nitrogen. After slices were collected from a complete experiment (i.e., slices from 4 to 6 mice per age, at P10 and P28), slices were homogenized by sonication in 1% SDS and prepared as described above for Western blot analysis.

### Patch-Clamp Electrophysiology

Recording pipettes were fabricated from borosilicate glass microcapillaries and had resistances of 4–6 MΩ when filled with internal solution (see below). Putative SPNs were visualized in corticostriatal slices with a SliceScope (Scientifica, United Kingdom) equipped with a 40 × 0.8 NA water-immersion objective (LUMPlanFLN, Olympus, United States) and Dodt contrast tube optics. Data were recorded with a MultiClamp 700B amplifier (Molecular Devices, United States), low-pass filtered at 5 kHz and digitized at 10 kHz with a digidata 1550b to a personal computer running pClamp 11 (Molecular Devices). Cell capacitance and series resistance (<25 MOhm) were left uncompensated but were monitored by applying a hyperpolarizing voltage-command (5 mV) at regular intervals. Recordings where series resistance increased above 25 MOhm were discarded.

Whole cell patch-clamp recordings of miniature excitatory and inhibitory synaptic currents (mEPSCs, mIPSCs, “minis”) were performed at −70 mV holding potential, in the presence of tetrodotoxin (TTX, 0.5 μM) and picrotoxin (100 μM, mEPSCs) or kynurenic acid (1 mM, mIPSCs). For mEPSCs the recording pipette contained (in mM): K-gluconate, 120; KCl, 20; EGTA, 0.1; HEPES, 10; Na_2_-phosphocreatine, 5; Na_2_ATP, 4; Na_2_GTP, 0.3, adjusted to pH 7.25 with KOH (300 mOsm/l), and for mIPSCs the pipettes contained (in mM): KCl, 140; HEPES, 10; EGTA, 1; Na_2_ATP, 2; Na_2_GTP 0.2; pH 7.25. Recordings were performed at 32–34°C (TC324C, Warner Instruments, United States).

The cells were filled with recording solution for at least 10 min and data was collected during 3 min. The amplitude and frequency of the minis were analyzed off-line using Clampfit 10.7. Traces were filtered at 1 kHz and synaptic events were detected using the event detection function and template search. The amplitude and frequency from each recording were averaged and group comparisons were performed between slices using unpaired *t*-test assuming equal variances. The number of mice were > 4 per group.

### Western Blot

Equivalent amount of protein per sample (5–25 μg/well) were loaded into 10, 12, or 4–12% gradient polyacrylamide gels as described (Santini et al., 2007). Protein was transferred from the gel to an Immobilon FL PVDF membrane (pore size 0.2 μm). Blots were blocked in TBS with 0.1% Tween-20 (TBST) and 5% fat-free, dry milk for 1 h at room temperature. Blots were then incubated with primary antibody (see Table 1 for detailed information regarding antibodies) diluted in TBST with 5% BSA as specified (see Table 1 for dilution and antibody sources) overnight at 4°C. Blots were then washed with TBST and incubated in secondary antibody for 1 h at room temperature. Blots were developed using either the Odyssey imaging system (LICOR) or an enhanced chemiluminescence (ECL) system (Amersham) and imaged using an Azure Biosystems C600 system. Western blots developed using the Odyssey system were analyzed in Image Studio Lite (LICOR). Western blots developed with the ECL system were analyzed using standard routines in ImageJ. All samples were probed for beta-actin and DARPP32 as loading and dissection controls, respectively.

**TABLE 1:** Antibodies.

Antibody	Company (Catalog No.)	Dilution	Detection method	Notes
Rabbit anti LC3B	Novus Biologicals (NB600-1384)	1:1000	Odyssey	12% SDS-PAGE gel required
Rabbit anti p62	MBL (PM045)	1:1000	Odyssey	Atg7KO validated (data not shown)
Rabbit anti Darpp32	Cell Signaling Technology (2306)	1:2500–5000	Odyssey	
Mouse anti β-Actin	Novus Biologicals (NB600-501)	1:5000–10000	Odyssey	
Rabbit anti p-ULK1 (Ser757)	Cell Signaling Technology (6888)	1:500	Odyssey	
Rabbit anti Beclin	Cell Signaling Technology (3495)	1:1000	ECL	
Rabbit anti p-Beclin-1 (Ser14)	Cell Signaling Technology (84966)	1:1000	ECL	
Rabbit anti S6 ribosomal protein	Cell Signaling Technology (2217)	1:1000–2000	ECL	
Rabbit anti p-S6 ribosomal protein (Ser240/244)	Cell Signaling Technology (2215)	1:1000–2000	ECL	
Rabbit anti p44/42 MAPK (Erk1/2)	Cell Signaling Technology (4695)	1:1000	Odyssey	
Rabbit anti p-p44/42 MAPK (Erk1/2; Thr202/Tyr204)	Cell Signaling Technology (4370)	1:1000	Odyssey	

### Drugs and Chemicals

Sodium valproate (VPA) was purchased from ACROS Organics, dissolved in saline solution and administered subcutaneously to pregnant females at the dose of 600 mg/kg. BafA1 and Torin-1 were purchased from Tocris. SAR 405 was purchased from Cayman Chemicals. Unless otherwise stated, all drugs were dissolved in DMSO and slices were incubated in ACSF containing the drugs or equivalent volume of vehicles for 3 h at 34°C. DMSO did not exceed a final concentration of 0.1%. All other chemicals were purchased from Fisher Scientific.

Bafilomycin A1 was used at a concentration of 100 nM, a concentration that effectively blocks autophagolysosome fusion in primary neuronal culture and in transformed cell lines (Yamamoto et al., 1998; Maday and Holzbaur, 2016; Redmann et al., 2017). SAR405 was used at 1 μM in acute brain slice experiments. This concentration showed both maximal efficacy in inhibiting autophagy and no off target effects in cell culture experiments (Ronan et al., 2014). Torin-1 was used at 5 μM. Rapamycin, another mTOR inhibitor is generally used at concentrations between 1 and 5 μM in both cell culture and in acute brain slice; however, Torin-1 has an IC50 of mTORC1 activity about 5X greater than the IC50 of rapamycin (Thoreen et al., 2009). Therefore, we used 5 μM and confirmed that this inhibited rpS6 phosphorylation and mTOR activity in the acute brain slice.

Rapamycin (LC Laboratories) was dissolved in 1% DMSO and 5% Tween-80 in saline and injected i.p. at the dose of 5 mg/kg (vehicle treated mice received an equivalent dose of vehicle). Rapamycin (5 mg/kg) was injected i.p. once a day for 5 days. On the last day of the treatment, the mice were subjected to the three chamber sociability test (see below) 1 h after the injection. This regimen was chosen because it generates brain rapamycin concentrations in a range previously established to inhibit mTOR and normalize ASD-like behaviors and behaviors dependent on mTOR activation (Ehninger et al., 2008; Zhou et al., 2009; Huynh et al., 2014; Tsai et al., 2018).

### 2p-Microscopy

Two-photon images were acquired on a Prairie Ultima microscope system (Middleton, WI, United States) using PrairieView 4.3 software. Acute brain slices were transferred into a chamber and perfused with oxygenated ACSF at room temperature. Samples were excited with a Coherent (Santa Clara, CA, United States) Chameleon Ultra two-photon laser at 980 nm, and images were simultaneously collected through two photomultiplier tube channels with corresponding 585–630 and 490–560 nm emission windows. The objective used was a 60X, 0.9 NA water immersion lens, and images were 1024 × 1024 pixels in size.

### Immunohistochemistry

Mice were deeply anesthestized and transcardially perfused with 0.9% NaCl followed by 4% paraformaldehyde (PFA) in 0.1M phosphate buffer (PB). Brains were removed and post-fixed overnight in 4% PFA in 0.1M PB. Brains were then washed three times in 1X phosphate buffered saline and cut into 40 μm sections using a VT1200 vibratome (Leica Biosystems) and stored in cryoprotectant (0.1M PB, 30% glycerol, 30% ethylene glycol) at −20°C. For immunofluorescence analysis, sections were washed in TBS three times and then blocked and permeabilized for 1 h at room temperature with 10% normal donkey serum (Jackson Immunoresearch) and 0.1% Triton-X in TBS. Sections were then incubated overnight at 4°C with primary antibodies in 2% normal donkey serum, 0.1% Triton-X in TBS. Primary antibodies included: Rabbit anti-DARPP32 (Cell Signaling), and Chicken anti-Green Fluorescent Protein (Abcam). Secondary antibodies with the appropriate conjugated fluorphores were purchased from Invitrogen. The endogenous fluorescence of RFP was imaged. Sections were then washed in TBS and mounted. Images were obtained using a Leica SP5 confocal system with argon, DPSS He/Ne lasers. Images were obtained with a 63X oil immersion objective with a 2X digital zoom at 2048 × 2048 resolution (∼120 nm resolution). All images were taken with the same laser intensity and detector settings with non-saturating pixel intensities.

Image analysis was conducted in ImageJ. Cell bodies were segmented using DARPP32 stained (DARPP32+) and not stained (DARPP32−) cells. RFP + puncta within each segment was counted manually. 10–20 cells were counted per section, 2–4 sections were counted per animal. Average number of puncta per cell was determined from *n* = 3 animals per age. All images were collected, and all analyses were conducted blind to condition.

### Marble Burying Test

Marble burying was measured as previously described (Arndt et al., 2005; Centonze et al., 2008; Shafritz et al., 2008; Shmelkov et al., 2010; Peça et al., 2011; Peñagarikano et al., 2011; Dölen et al., 2013; Santini et al., 2013). Briefly, 20 glass marbles (around 15 mm in diameter) were arranged in a symmetrical (4 × 5) grid on the surface of 2–3 cm deep bedding in clean, standard mouse cages with transparent wall extensions (20 cm) to avoid climbing or jumping of mice. Each mouse was placed in the center of the cage for a 20-min exploration period, after which the number of marbles buried was tallied by investigators blind to the treatment. ‘Buried’ was defined as > 50% covered by bedding according to Thomas et al. (2009). Testing was performed under dim light (∼15 lux) and recorded with a videocamera.

### Three Chamber Sociability Test

Social behavior was measured using a 3-chambered social arena as described previously (Moy et al., 2008). Briefly, mice received a 10 min habituation session to the arena in the presence of two wire cages, one in each side of the chamber. A 10 min social preference test followed in which the mice were allowed to explore the arena containing a wired cage with a conspecific mouse (stranger mouse) or a wire cage with an inanimate object in the two lateral chambers. The placement of social and non-social targets was counterbalanced between animals. The measured parameters of social interaction and locomotor activity were the interaction time (sec) with each target (during the social preference test session) and the total distance (cm) moved (during habituation and social preference test sessions) respectively, calculated by Ethovision XT video tracking software (Noldus).

### Statistical Analysis

All analysis was conducted blind to condition. For statistical analysis between two groups, unpaired, two-tailed *t*-tests were used. For analysis between three or more groups, one-way ANOVA was used. Sociability test was analyzed with two-way repeated measure ANOVA (within factors: treatment and social cue), followed by multiple comparisons using Bonferroni’s test. Normality was not formally tested. Sample size was not based on a formal power analysis but was based on past work from our groups and similar experiments from the literature. Statistical analysis was conducted in GraphPad Prism 7 (La Jolla, CA, United States). All bar graphs show the mean ± SEM.

## Data Availability Statement

The datasets generated for this study are available on request to the corresponding author.

## Ethics Statement

The animal study was reviewed and approved by Columbia University Institutional Animal Care and Use Committee.

## Author Contributions

OL and ES: conception. OL, MP, AB, and ES: methodology. OL, IP, VC, MM, MB, MP, JC, AB, and ES: investigation. OL: writing – original draft and visualization. OL, IP, MP, DS, AB, and ES: writing – review and editing. ES: supervision. DS and ES: funding acquisition.

## Conflict of Interest

The authors declare that the research was conducted in the absence of any commercial or financial relationships that could be construed as a potential conflict of interest.

## References

[B1] Arndt T. L. Stodgell C. J. Rodier P. M. (2005). The teratology of autism. *Int. J. Dev. Neurosci.* 23 189–199. 10.1016/j.ijdevneu.2004.11.001 15749245

[B2] Auerbach B. D. Osterweil E. K. Bear M. F. (2011). Mutations causing syndromic autism define an axis of synaptic pathophysiology. *Nature* 480 63–68. 10.1038/nature10658 22113615 PMC3228874

[B3] Bagni C. Zukin R. S. (2019). A synaptic perspective of fragile X syndrome and autism spectrum disorders. *Neuron* 101 1070–1088. 10.1016/j.neuron.2019.02.041 30897358 PMC9628679

[B4] Belandia B. Brautigan D. Martín-Pérez J. (1994). Attenuation of ribosomal protein S6 phosphatase activity in chicken embryo fibroblasts transformed by Rous sarcoma virus. *Mol. Cell. Biol.* 14 200–206. 10.1128/mcb.14.1.200 8264587 PMC358370

[B5] Bento C. F. Renna M. Ghislat G. Puri C. Ashkenazi A. Vicinanza M. (2016). Mammalian autophagy: how does it work? *Annu. Rev. Biochem.* 85 685–713. 10.1146/annurev-biochem-060815-14556 26865532

[B6] Bockaert J. Marin P. (2015). mTOR in brain physiology and pathologies. *Physiol. Rev.* 95 1157–1187. 10.1152/physrev.00038.2014 26269525

[B7] Bonito-Oliva A. Pallottino S. Bertran-Gonzalez J. Girault J.-A. Valjent E. Fisone G. (2013). Haloperidol promotes mTORC1-dependent phosphorylation of ribosomal protein S6 via dopamine- and cAMP-regulated phosphoprotein of 32 kDa and inhibition of protein phosphatase-1. *Neuropharmacology* 72 197–203. 10.1016/j.neuropharm.2013.04.043 23643747

[B8] Bromley R. L. Mawer G. E. Briggs M. Cheyne C. Clayton-Smith J. García-Fiñana M. (2013). The prevalence of neurodevelopmental disorders in children prenatally exposed to antiepileptic drugs. *J. Neurol. Neurosurg. Psychiatry* 84 637–643. 10.1136/jnnp-2012-304270 23370617 PMC4115188

[B9] Byrne S. Jansen L. U-King-Im J.-M. Siddiqui A. Lidov H. G. W. Bodi I. (2016). EPG5-related Vici syndrome: a paradigm of neurodevelopmental disorders with defective autophagy. *Brain* 139 765–781. 10.1093/brain/awv393 26917586 PMC4766378

[B10] Cartocci V. Catallo M. Tempestilli M. Segatto M. Pfrieger F. W. Bronzuoli M. R. (2018). Altered brain cholesterol/isoprenoid metabolism in a rat model of autism spectrum disorders. *Neuroscience* 372 27–37. 10.1016/j.neuroscience.2017.12.053 29309878

[B11] Centonze D. Rossi S. Mercaldo V. Napoli I. Ciotti M. T. De Chiara V. (2008). Abnormal striatal GABA transmission in the mouse model for the fragile X syndrome. *Biol. Psychiatry* 63 963–973. 10.1016/j.biopsych.2007.09.008 18028882

[B12] Chakrabarti L. Eng J. Ivanov N. Garden G. A. La Spada A. R. (2009). Autophagy activation and enhanced mitophagy characterize the Purkinje cells of pcd mice prior to neuronal death. *Mol. Brain* 2:24. 10.1186/1756-6606-2-24 19640278 PMC2729476

[B13] Chomiak T. Turner N. Hu B. (2013). What we have learned about autism spectrum disorder from valproic acid. *Patholog. Res. Int.* 2013:712758. 10.1155/2013/712758 24381784 PMC3871912

[B14] Christensen J. Grønborg T. K. Sørensen M. J. Schendel D. Parner E. T. Pedersen L. H. (2013). Prenatal valproate exposure and risk of autism spectrum disorders and childhood autism. *JAMA* 309 1696–1703. 10.1001/jama.2013.2270 23613074 PMC4511955

[B15] Curatolo P. Franz D. N. Lawson J. A. Yapici Z. Ikeda H. Polster T. (2018). Adjunctive everolimus for children and adolescents with treatment-refractory seizures associated with tuberous sclerosis complex: post-hoc analysis of the phase 3 EXIST-3 trial. *Lancet Child Adolesc. Health* 2 495–504. 10.1016/S2352-4642(18)30099-30093 30169322

[B16] Delmonte S. Balsters J. H. McGrath J. Fitzgerald J. Brennan S. Fagan A. J. (2012). Social and monetary reward processing in autism spectrum disorders. *Mol. Autism* 3:7. 10.1186/2040-2392-3-7 23014171 PMC3499449

[B17] Diagnostic and Statistical Manual of Mental Disorders DSM-5^®^ (2013). Available online at: https://www.appi.org/Diagnostic_and_Statistical_Manual_of_Mental_Disorders_DSM-5_Fifth_Edition (Accessed July 25, 2019). 10.1590/s2317-17822013000200017 24413388

[B18] Dölen G. Darvishzadeh A. Huang K. W. Malenka R. C. (2013). Social reward requires coordinated activity of nucleus accumbens oxytocin and serotonin. *Nature* 501 179–184. 10.1038/nature12518 24025838 PMC4091761

[B19] Dragich J. M. Kuwajima T. Hirose-Ikeda M. Yoon M. S. Eenjes E. Bosco J. R. (2016). Autophagy linked FYVE (Alfy/WDFY3) is required for establishing neuronal connectivity in the mammalian brain. *eLife* 5:e14810. 10.7554/eLife.14810 27648578 PMC5030082

[B20] Ebrahimi-Fakhari D. Saffari A. Wahlster L. Di Nardo A. Turner D. Lewis T. L. (2016). Impaired mitochondrial dynamics and mitophagy in neuronal models of tuberous sclerosis complex. *Cell Rep.* 17 1053–1070. 10.1016/j.celrep.2016.09.054 27760312 PMC5078873

[B21] Ehninger D. Han S. Shilyansky C. Zhou Y. Li W. Kwiatkowski D. J. (2008). Reversal of learning deficits in a Tsc2± mouse model of tuberous sclerosis. *Nat. Med.* 14 843–848. 10.1038/nm1788 18568033 PMC2664098

[B22] Ferrari D. C. Mdzomba B. J. Dehorter N. Lopez C. Michel F. J. Libersat F. (2012). Midbrain dopaminergic neurons generate calcium and sodium currents and release dopamine in the striatum of pups. *Front. Cell Neurosci.* 6:7. 10.3389/fncel.2012.00007 22408606 PMC3297358

[B23] Fox J. H. Connor T. Chopra V. Dorsey K. Kama J. A. Bleckmann D. (2010). The mTOR kinase inhibitor Everolimus decreases S6 kinase phosphorylation but fails to reduce mutant huntingtin levels in brain and is not neuroprotective in the R6/2 mouse model of Huntington’s disease. *Mol. Neurodegener.* 5:26. 10.1186/1750-1326-5-26 20569486 PMC2908080

[B24] French J. A. Lawson J. A. Yapici Z. Ikeda H. Polster T. Nabbout R. (2016). Adjunctive everolimus therapy for treatment-resistant focal-onset seizures associated with tuberous sclerosis (EXIST-3): a phase 3, randomised, double-blind, placebo-controlled study. *Lancet* 388 2153–2163. 10.1016/S0140-6736(16)31419-31412 27613521

[B25] Fuccillo M. V. (2016). Striatal circuits as a common node for autism pathophysiology. *Front. Neurosci.* 10:27 10.3389/fnins.2016.00027 PMC4746330 26903795

[B26] Gerfen C. R. Surmeier D. J. (2011). Modulation of striatal projection systems by dopamine. *Annu. Rev. Neurosci.* 34 441–466. 10.1146/annurev-neuro-061010-113641 21469956 PMC3487690

[B27] Gkogkas C. G. Khoutorsky A. Ran I. Rampakakis E. Nevarko T. Weatherill D. B. (2013). Autism-related deficits via dysregulated eIF4E-dependent translational control. *Nature* 493 371–377. 10.1038/nature11628 23172145 PMC4133997

[B28] González-Polo R.-A. Boya P. Pauleau A.-L. Jalil A. Larochette N. Souquère S. (2005). The apoptosis/autophagy paradox: autophagic vacuolization before apoptotic death. *J. Cell Sci.* 118 3091–3102. 10.1242/jcs.02447 15985464

[B29] Hara T. Nakamura K. Matsui M. Yamamoto A. Nakahara Y. Suzuki-Migishima R. (2006). Suppression of basal autophagy in neural cells causes neurodegenerative disease in mice. *Nature* 441 885–889. 10.1038/nature04724 16625204

[B30] He C. Klionsky D. J. (2009). Regulation mechanisms and signaling pathways of autophagy. *Annu. Rev. Genet.* 43 67–93. 10.1146/annurev-genet-102808-114910 19653858 PMC2831538

[B31] Henderson C. Wijetunge L. Kinoshita M. N. Shumway M. Hammond R. S. Postma F. R. (2012). Reversal of disease-related pathologies in the fragile X mouse model by selective activation of GABAB receptors with arbaclofen. *Sci. Transl. Med.* 4:152ra128. 10.1126/scitranslmed.3004218 22993295 PMC8826584

[B32] Hernandez D. Torres C. A. Setlik W. Cebrián C. Mosharov E. V. Tang G. (2012). Regulation of presynaptic neurotransmission by macroautophagy. *Neuron* 74 277–284. 10.1016/j.neuron.2012.02.020 22542182 PMC3578406

[B33] Hor C. H. H. Tang B. L. (2018). Beta-propeller protein-associated neurodegeneration (BPAN) as a genetically simple model of multifaceted neuropathology resulting from defects in autophagy. *Rev. Neurosci.* 30 261–277. 10.1515/revneuro-2018-2045 30204590

[B34] Huynh T. N. Santini E. Klann E. (2014). Requirement of Mammalian target of rapamycin complex 1 downstream effectors in cued fear memory reconsolidation and its persistence. *J. Neurosci.* 34 9034–9039. 10.1523/JNEUROSCI.0878-14.2014 24990923 PMC4078080

[B35] Huynh T. N. Shah M. Koo S. Y. Faraud K. S. Santini E. Klann E. (2015). eIF4E/Fmr1 double mutant mice display cognitive impairment in addition to ASD-like behaviors. *Neurobiol. Dis.* 83 67–74. 10.1016/j.nbd.2015.08.016 26306459 PMC4674395

[B36] Itakura E. Kishi C. Inoue K. Mizushima N. (2008). Beclin 1 forms two distinct phosphatidylinositol 3-kinase complexes with mammalian Atg14 and UVRAG. *Mol. Biol. Cell* 19 5360–5372. 10.1091/mbc.E08-01-0080 18843052 PMC2592660

[B37] Jentink J. Loane M. A. Dolk H. Barisic I. Garne E. Morris J. K. (2010). Valproic acid monotherapy in pregnancy and major congenital malformations. *N. Engl. J. Med.* 362 2185–2193. 10.1056/NEJMoa0907328 20558369

[B38] Jung C. H. Jun C. B. Ro S.-H. Kim Y.-M. Otto N. M. Cao J. (2009). ULK-Atg13-FIP200 complexes mediate mTOR signaling to the autophagy machinery. *Mol. Biol. Cell* 20 1992–2003. 10.1091/mbc.E08-12-1249 19225151 PMC2663920

[B39] Kabeya Y. Mizushima N. Ueno T. Yamamoto A. Kirisako T. Noda T. (2000). LC3, a mammalian homologue of yeast Apg8p, is localized in autophagosome membranes after processing. *EMBO J.* 19 5720–5728. 10.1093/emboj/19.21.5720 11060023 PMC305793

[B40] Kaushik S. Rodriguez-Navarro J. A. Arias E. Kiffin R. Sahu S. Schwartz G. J. (2011). Autophagy in hypothalamic AgRP neurons regulates food intake and energy balance. *Cell Metab.* 14, 173–183. 10.1016/j.cmet.2011.06.008 21803288 PMC3148494

[B41] Kim H. J. Cho M. H. Shim W. H. Kim J. K. Jeon E. Y. Kim D. H. (2017). Deficient autophagy in microglia impairs synaptic pruning and causes social behavioral defects. *Mol. Psychiatry* 22 1576–1584. 10.1038/mp.2016.103 27400854 PMC5658669

[B42] Kim J. Kundu M. Viollet B. Guan K.-L. (2011). AMPK and mTOR regulate autophagy through direct phosphorylation of Ulk1. *Nat. Cell Biol.* 13 132–141. 10.1038/ncb2152 21258367 PMC3987946

[B43] Klionsky D. J. Abdelmohsen K. Abe A. Abedin M. J. Abeliovich H. Acevedo Arozena A. (2016). Guidelines for the use and interpretation of assays for monitoring autophagy (3rd edition). *Autophagy* 12 1–222. 10.1080/15548627.2015.1100356 26799652 PMC4835977

[B44] Komatsu M. Waguri S. Chiba T. Murata S. Iwata J. Tanida I. (2006). Loss of autophagy in the central nervous system causes neurodegeneration in mice. *Nature* 441 880–884. 10.1038/nature04723 16625205

[B45] Kuo H.-Y. Liu F.-C. (2017). Valproic acid induces aberrant development of striatal compartments and corticostriatal pathways in a mouse model of autism spectrum disorder. *FASEB J.* 31 4458–4471. 10.1096/fj.201700054R 28687613

[B46] Kwon C.-H. Luikart B. W. Powell C. M. Zhou J. Matheny S. A. Zhang W. (2006). Pten regulates neuronal arborization and social interaction in mice. *Neuron* 50 377–388. 10.1016/j.neuron.2006.03.023 16675393 PMC3902853

[B47] Langen M. Durston S. Staal W. G. Palmen S. J. M. C. van Engeland H. (2007). Caudate nucleus is enlarged in high-functioning medication-naive subjects with autism. *Biol. Psychiatry* 62 262–266. 10.1016/j.biopsych.2006.09.040 17224135

[B48] Langen M. Schnack H. G. Nederveen H. Bos D. Lahuis B. E. de Jonge M. V. (2009). Changes in the developmental trajectories of striatum in autism. *Biol. Psychiatry* 66 327–333. 10.1016/j.biopsych.2009.03.017 19423078

[B49] Lauber E. Filice F. Schwaller B. (2016). Prenatal valproate exposure differentially affects parvalbumin-expressing neurons and related circuits in the cortex and striatum of mice. *Front. Mol. Neurosci.* 9:150 10.3389/fnmol.2016.00150 PMC5174119 28066177

[B50] Lee K.-M. Hwang S.-K. Lee J.-A. (2013). Neuronal autophagy and neurodevelopmental disorders. *Exp. Neurobiol.* 22 133–142. 10.5607/en.2013.22.3.133 24167408 PMC3807000

[B51] Li L. Wang Z. V. Hill J. A. Lin F. (2014). New autophagy reporter mice reveal dynamics of proximal tubular autophagy. *J. Am. Soc. Nephrol.* 25 305–315. 10.1681/ASN.2013040374 24179166 PMC3904563

[B52] Lieberman O. J. Frier M. D. McGuirt A. F. Griffey C. J. Rafikian E. Yang M. (2020). Cell-type-specific regulation of neuronal intrinsic excitability by macroautophagy. *eLife* 9:e50843. 10.7554/eLife.50843 31913125 PMC6984822

[B53] Lieberman O. J. McGuirt A. F. Mosharov E. V. Pigulevskiy I. Hobson B. D. Choi S. (2018). Dopamine triggers the maturation of striatal spiny projection neuron excitability during a critical period. *Neuron* 99 540.e4–554.e4. 10.1016/j.neuron.2018.06.044 30057204 PMC6602586

[B54] Lieberman O. J. McGuirt A. F. Tang G. Sulzer D. (2019a). Roles for neuronal and glial autophagy in synaptic pruning during development. *Neurobiol. Dis.* 122 49–63. 10.1016/j.nbd.2018.04.017 29709573 PMC6204314

[B55] Lieberman O. J. Pigulevskiy I. Post M. R. Sulzer D. Santini E. (2019b). mTOR suppresses macroautophagy during postnatal development of the striatum. *bioRxiv* [Preprint]. 10.1101/536680 PMC7136750 32296308

[B56] Maday S. Holzbaur E. L. F. (2016). Compartment-Specific Regulation of Autophagy in Primary Neurons. *J. Neurosci.* 36 5933–5945. 10.1523/JNEUROSCI.4401-15.2016 27251616 PMC4887563

[B57] Magnuson B. Ekim B. Fingar D. C. (2012). Regulation and function of ribosomal protein S6 kinase (S6K) within mTOR signalling networks. *Biochem. J.* 441 1–21. 10.1042/BJ20110892 22168436

[B58] McMahon J. Huang X. Yang J. Komatsu M. Yue Z. Qian J. (2012). Impaired autophagy in neurons after disinhibition of mammalian target of rapamycin and its contribution to epileptogenesis. *J. Neurosci.* 32 15704–15714. 10.1523/JNEUROSCI.2392-12.2012 23136410 PMC3501684

[B59] Meador K. J. Baker G. A. Browning N. Cohen M. J. Bromley R. L. Clayton-Smith J. (2013). Fetal antiepileptic drug exposure and cognitive outcomes at age 6 years (NEAD study): a prospective observational study. *Lancet Neurol.* 12 244–252. 10.1016/S1474-4422(12)70323-X 23352199 PMC3684942

[B60] Mizushima N. Yamamoto A. Matsui M. Yoshimori T. Ohsumi Y. (2004). In vivo analysis of autophagy in response to nutrient starvation using transgenic mice expressing a fluorescent autophagosome marker. *Mol. Biol. Cell* 15, 1101–1111. 10.1091/mbc.e03-09-0704 14699058 PMC363084

[B61] Moy S. S. Nadler J. J. Poe M. D. Nonneman R. J. Young N. B. Koller B. H. (2008). Development of a mouse test for repetitive, restricted behaviors: relevance to autism. *Behav. Brain Res.* 188 178–194. 10.1016/j.bbr.2007.10.029 18068825 PMC2349090

[B62] Nicolini C. Fahnestock M. (2018). The valproic acid-induced rodent model of autism. *Exp. Neurol.* 299 217–227. 10.1016/j.expneurol.2017.04.017 28472621

[B63] Nikoletopoulou V. Sidiropoulou K. Kallergi E. Dalezios Y. Tavernarakis N. (2017). Modulation of autophagy by BDNF underlies synaptic plasticity. *Cell Metab.* 26 230.e5–242.e5. 10.1016/j.cmet.2017.06.005 28683289

[B64] Ohsumi Y. (2014). Historical landmarks of autophagy research. *Cell Res.* 24 9–23. 10.1038/cr.2013.169 24366340 PMC3879711

[B65] Peça J. Feliciano C. Ting J. T. Wang W. Wells M. F. Venkatraman T. N. (2011). Shank3 mutant mice display autistic-like behaviours and striatal dysfunction. *Nature* 472 437–442. 10.1038/nature09965 21423165 PMC3090611

[B66] Peixoto R. T. Wang W. Croney D. M. Kozorovitskiy Y. Sabatini B. L. (2016). Early hyperactivity and precocious maturation of corticostriatal circuits in Shank3B(-/-) mice. *Nat. Neurosci.* 19 716–724. 10.1038/nn.4260 26928064 PMC4846490

[B67] Peñagarikano O. Abrahams B. S. Herman E. I. Winden K. D. Gdalyahu A. Dong H. (2011). Absence of CNTNAP2 leads to epilepsy, neuronal migration abnormalities, and core autism-related deficits. *Cell* 147 235–246. 10.1016/j.cell.2011.08.040 21962519 PMC3390029

[B68] Plotkin J. L. Wu N. Chesselet M.-F. Levine M. S. (2005). Functional and molecular development of striatal fast-spiking GABAergic interneurons and their cortical inputs. *Eur. J. Neurosci.* 22 1097–1108. 10.1111/j.1460-9568.2005.04303.x 16176351

[B69] Poultney C. S. Goldberg A. P. Drapeau E. Kou Y. Harony-Nicolas H. Kajiwara Y. (2013). Identification of small exonic CNV from whole-exome sequence data and application to autism spectrum disorder. *Am. J. Hum. Genet.* 93 607–619. 10.1016/j.ajhg.2013.09.001 24094742 PMC3791269

[B70] Qin L. Dai X. Yin Y. (2016). Valproic acid exposure sequentially activates Wnt and mTOR pathways in rats. *Mol. Cell. Neurosci.* 75 27–35. 10.1016/j.mcn.2016.06.004 27343825

[B71] Redmann M. Benavides G. A. Berryhill T. F. Wani W. Y. Ouyang X. Johnson M. S. (2017). Inhibition of autophagy with bafilomycin and chloroquine decreases mitochondrial quality and bioenergetic function in primary neurons. *Redox Biol.* 11 73–81. 10.1016/j.redox.2016.11.004 27889640 PMC5124357

[B72] Ronan B. Flamand O. Vescovi L. Dureuil C. Durand L. Fassy F. (2014). A highly potent and selective Vps34 inhibitor alters vesicle trafficking and autophagy. *Nat. Chem. Biol.* 10 1013–1019. 10.1038/nchembio.1681 25326666

[B73] Roullet F. I. Lai J. K. Y. Foster J. A. (2013). In utero exposure to valproic acid and autism–a current review of clinical and animal studies. *Neurotoxicol. Teratol.* 36 47–56. 10.1016/j.ntt.2013.01.004 23395807

[B74] Rowland A. M. Richmond J. E. Olsen J. G. Hall D. H. Bamber B. A. (2006). Presynaptic terminals independently regulate synaptic clustering and autophagy of GABAA receptors in *Caenorhabditis elegans*. *J. Neurosci.* 26 1711–1720. 10.1523/JNEUROSCI.2279-05.2006 16467519 PMC6793639

[B75] Russell R. C. Tian Y. Yuan H. Park H. W. Chang Y.-Y. Kim J. (2013). ULK1 induces autophagy by phosphorylating Beclin-1 and activating VPS34 lipid kinase. *Nat. Cell Biol.* 15 741–750. 10.1038/ncb2757 23685627 PMC3885611

[B76] Santini E. Heiman M. Greengard P. Valjent E. Fisone G. (2009). Inhibition of mTOR signaling in Parkinson’s disease prevents L-DOPA-induced dyskinesia. *Sci. Signal.* 2:ra36. 10.1126/scisignal.2000308 19622833

[B77] Santini E. Huynh T. N. MacAskill A. F. Carter A. G. Pierre P. Ruggero D. (2013). Exaggerated translation causes synaptic and behavioural aberrations associated with autism. *Nature* 493 411–415. 10.1038/nature11782 23263185 PMC3548017

[B78] Santini E. Valjent E. Usiello A. Carta M. Borgkvist A. Girault J.-A. (2007). Critical involvement of cAMP/DARPP-32 and extracellular signal-regulated protein kinase signaling in L-DOPA-induced dyskinesia. *J. Neurosci.* 27 6995–7005. 10.1523/JNEUROSCI.0852-07.2007 17596448 PMC6672217

[B79] Sears L. L. Vest C. Mohamed S. Bailey J. Ranson B. J. Piven J. (1999). An MRI study of the basal ganglia in autism. *Prog. Neuropsychopharmacol. Biol. Psychiatry* 23 613–624. 10.1016/S0278-5846(99)00020-22 10390720

[B80] Shafritz K. M. Dichter G. S. Baranek G. T. Belger A. (2008). The neural circuitry mediating shifts in behavioral response and cognitive set in autism. *Biol. Psychiatry* 63 974–980. 10.1016/j.biopsych.2007.06.028 17916328 PMC2599927

[B81] Shehata M. Matsumura H. Okubo-Suzuki R. Ohkawa N. Inokuchi K. (2012). Neuronal stimulation induces autophagy in hippocampal neurons that is involved in AMPA receptor degradation after chemical long-term depression. *J. Neurosci.* 32 10413–10422. 10.1523/JNEUROSCI.4533-11.2012 22836274 PMC6703735

[B82] Shen W. Ganetzky B. (2009). Autophagy promotes synapse development in *Drosophila*. *J. Cell Biol.* 187 71–79. 10.1083/jcb.200907109 19786572 PMC2762098

[B83] Shmelkov S. V. Hormigo A. Jing D. Proenca C. C. Bath K. G. Milde T. (2010). Slitrk5 deficiency impairs corticostriatal circuitry and leads to obsessive-compulsive-like behaviors in mice. *Nat. Med.* 16 598–602. 10.1038/nm.2125 20418887 PMC2907076

[B84] Shpilka T. Weidberg H. Pietrokovski S. Elazar Z. (2011). Atg8: an autophagy-related ubiquitin-like protein family. *Genome Biol.* 12:226. 10.1186/gb-2011-12-7-226 21867568 PMC3218822

[B85] Silverman J. L. Smith D. G. Rizzo S. J. S. Karras M. N. Turner S. M. Tolu S. S. (2012). Negative allosteric modulation of the mGluR5 receptor reduces repetitive behaviors and rescues social deficits in mouse models of autism. *Sci. Transl. Med.* 4:131ra51. 10.1126/scitranslmed.3003501 22539775 PMC4904784

[B86] Song D. D. Harlan R. E. (1994). Genesis and migration patterns of neurons forming the patch and matrix compartments of the rat striatum. *Brain Res. Dev. Brain Res.* 83 233–245. 10.1016/0165-3806(94)00144-148 7535203

[B87] Stavoe A. K. H. Hill S. E. Hall D. H. Colón-Ramos D. A. (2016). KIF1A/UNC-104 transports atg-9 to regulate neurodevelopment and autophagy at synapses. *Dev. Cell* 38 171–185. 10.1016/j.devcel.2016.06.012 27396362 PMC4961624

[B88] Sutton L. P. Caron M. G. (2015). Essential role of D1R in the regulation of mTOR complex1 signaling induced by cocaine. *Neuropharmacology* 99 610–619. 10.1016/j.neuropharm.2015.08.024 26314207 PMC4703076

[B89] Tang G. Gudsnuk K. Kuo S.-H. Cotrina M. L. Rosoklija G. Sosunov A. (2014). Loss of mTOR-dependent macroautophagy causes autistic-like synaptic pruning deficits. *Neuron* 83 1131–1143. 10.1016/j.neuron.2014.07.040 25155956 PMC4159743

[B90] Tanida I. Minematsu-Ikeguchi N. Ueno T. Kominami E. (2005). Lysosomal turnover, but not a cellular level, of endogenous LC3 is a marker for autophagy. *Autophagy* 1 84–91. 10.4161/auto.1.2.1697 16874052

[B91] Tepper J. M. Sharpe N. A. Koós T. Z. Trent F. (1998). Postnatal development of the rat neostriatum: electrophysiological, light- and electron-microscopic studies. *Dev. Neurosci.* 20 125–145. 10.1159/000017308 9691188

[B92] Thomas A. Burant A. Bui N. Graham D. Yuva-Paylor L. A. Paylor R. (2009). Marble burying reflects a repetitive and perseverative behavior more than novelty-induced anxiety. *Psychopharmacology* 204 361–373. 10.1007/s00213-009-1466-y 19189082 PMC2899706

[B93] Thomas A. M. Bui N. Perkins J. R. Yuva-Paylor L. A. Paylor R. (2012). Group I metabotropic glutamate receptor antagonists alter select behaviors in a mouse model for fragile X syndrome. *Psychopharmacology* 219 47–58. 10.1007/s00213-011-2375-2374 21656124

[B94] Thoreen C. C. Kang S. A. Chang J. W. Liu Q. Zhang J. Gao Y. (2009). An ATP-competitive mammalian target of rapamycin inhibitor reveals rapamycin-resistant functions of mTORC1. *J. Biol. Chem.* 284 8023–8032. 10.1074/jbc.M900301200 19150980 PMC2658096

[B95] Tsai P. T. Hull C. Chu Y. Greene-Colozzi E. Sadowski A. R. Leech J. M. (2012). Autistic-like behaviour and cerebellar dysfunction in Purkinje cell Tsc1 mutant mice. *Nature* 488 647–651. 10.1038/nature11310 22763451 PMC3615424

[B96] Tsai P. T. Rudolph S. Guo C. Ellegood J. Gibson J. M. Schaeffer S. M. (2018). Sensitive periods for cerebellar-mediated autistic-like behaviors. *Cell Rep.* 25 357.e4–367.e4. 10.1016/j.celrep.2018.09.039 30304677 PMC6226056

[B97] Tsvetkov A. S. Miller J. Arrasate M. Wong J. S. Pleiss M. A. Finkbeiner S. (2010). A small-molecule scaffold induces autophagy in primary neurons and protects against toxicity in a Huntington disease model. *Proc. Natl. Acad. Sci. U.S.A.* 107 16982–16987. 10.1073/pnas.1004498107 20833817 PMC2947884

[B98] Voorn P. Kalsbeek A. Jorritsma-Byham B. Groenewegen H. J. (1988). The pre- and postnatal development of the dopaminergic cell groups in the ventral mesencephalon and the dopaminergic innervation of the striatum of the rat. *Neuroscience* 25 857–887. 10.1016/0306-4522(88)90041-90043 3405431

[B99] Wang Q. J. Ding Y. Kohtz D. S. Mizushima N. Cristea I. M. Rout M. P. (2006). Induction of autophagy in axonal dystrophy and degeneration. *J. Neurosci.* 26 8057–8068. 10.1523/JNEUROSCI.2261-06.2006 16885219 PMC6673783

[B100] Wang R. Tan J. Guo J. Zheng Y. Han Q. So K.-F. (2018). Aberrant development and synaptic transmission of cerebellar cortex in a VPA induced mouse autism model. *Front. Cell Neurosci.* 12:500. 10.3389/fncel.2018.00500 30622458 PMC6308145

[B101] Werler M. M. Ahrens K. A. Bosco J. L. F. Mitchell A. A. Anderka M. T. Gilboa S. M. (2011). Use of antiepileptic medications in pregnancy in relation to risks of birth defects. *Ann. Epidemiol.* 21 842–850. 10.1016/j.annepidem.2011.08.002 21982488 PMC4816042

[B102] Wu H. Zhang Q. Gao J. Sun C. Wang J. Xia W. (2018). Modulation of sphingosine 1-phosphate (S1P) attenuates spatial learning and memory impairments in the valproic acid rat model of autism. *Psychopharmacology* 235 873–886. 10.1007/s00213-017-4805-4804 29218394

[B103] Yamamoto A. Cremona M. L. Rothman J. E. (2006). Autophagy-mediated clearance of huntingtin aggregates triggered by the insulin-signaling pathway. *J. Cell Biol.* 172 719–731. 10.1083/jcb.200510065 16505167 PMC2063704

[B104] Yamamoto A. Simonsen A. (2011). The elimination of accumulated and aggregated proteins: a role for aggrephagy in neurodegeneration. *Neurobiol. Dis.* 43 17–28. 10.1016/j.nbd.2010.08.015 20732422 PMC2998573

[B105] Yamamoto A. Tagawa Y. Yoshimori T. Moriyama Y. Masaki R. Tashiro Y. (1998). Bafilomycin A1 prevents maturation of autophagic vacuoles by inhibiting fusion between autophagosomes and lysosomes in rat hepatoma cell line, H-4-II-E cells. *Cell Struct. Funct.* 23 33–42. 10.1247/csf.23.33 9639028

[B106] Yamamoto A. Yue Z. (2014). Autophagy and its normal and pathogenic states in the brain. *Annu. Rev. Neurosci.* 37 55–78. 10.1146/annurev-neuro-071013-14149 24821313

[B107] Yan J. Porch M. W. Court-Vazquez B. Bennett M. V. L. Zukin R. S. (2018). Activation of autophagy rescues synaptic and cognitive deficits in fragile X mice. *Proc. Natl. Acad. Sci. U.S.A.* 115 E9707–E9716. 10.1073/pnas.1808247115 30242133 PMC6187122

[B108] Yang D.-S. Stavrides P. Mohan P. S. Kaushik S. Kumar A. Ohno M. (2011). Reversal of autophagy dysfunction in the TgCRND8 mouse model of Alzheimer’s disease ameliorates amyloid pathologies and memory deficits. *Brain* 134 258–277. 10.1093/brain/awq341 21186265 PMC3009842

[B109] Yin H. H. Davis M. I. Ronesi J. A. Lovinger D. M. (2006). The role of protein synthesis in striatal long-term depression. *J. Neurosci.* 26 11811–11820. 10.1523/JNEUROSCI.3196-06.2006 17108154 PMC6674864

[B110] Zhang J. Zhang J.-X. Zhang Q.-L. (2016). PI3K/AKT/mTOR-mediated autophagy in the development of autism spectrum disorder. *Brain Res. Bull.* 125 152–158. 10.1016/j.brainresbull.2016.06.007 27320472

[B111] Zhang Y. Xiang Z. Jia Y. He X. Wang L. Cui W. (2019). The Notch signaling pathway inhibitor Dapt alleviates autism-like behavior, autophagy and dendritic spine density abnormalities in a valproic acid-induced animal model of autism. *Prog. Neuropsychopharmacol. Biol. Psychiatry* 94:109644. 10.1016/j.pnpbp.2019.109644 31075347

[B112] Zhou J. Blundell J. Ogawa S. Kwon C.-H. Zhang W. Sinton C. (2009). Pharmacological inhibition of mTORC1 suppresses anatomical, cellular, and behavioral abnormalities in neural-specific Pten knock-out mice. *J. Neurosci.* 29 1773–1783. 10.1523/JNEUROSCI.5685-08.2009 19211884 PMC3904448

