# Supplementary material for: mTOR Suppresses Macroautophagy During Striatal Postnatal Development and Is Hyperactive in Mouse Models of Autism Spectrum Disorders
Source: Front Cell Neurosci. 2020 Mar 31;14:70. doi: 10.3389/fncel.2020.00070 (PMC7136750; doi:10.3389/fncel.2020.00070)
Supplement: Supplementary file 1 [file Data_Sheet_1.PDF]

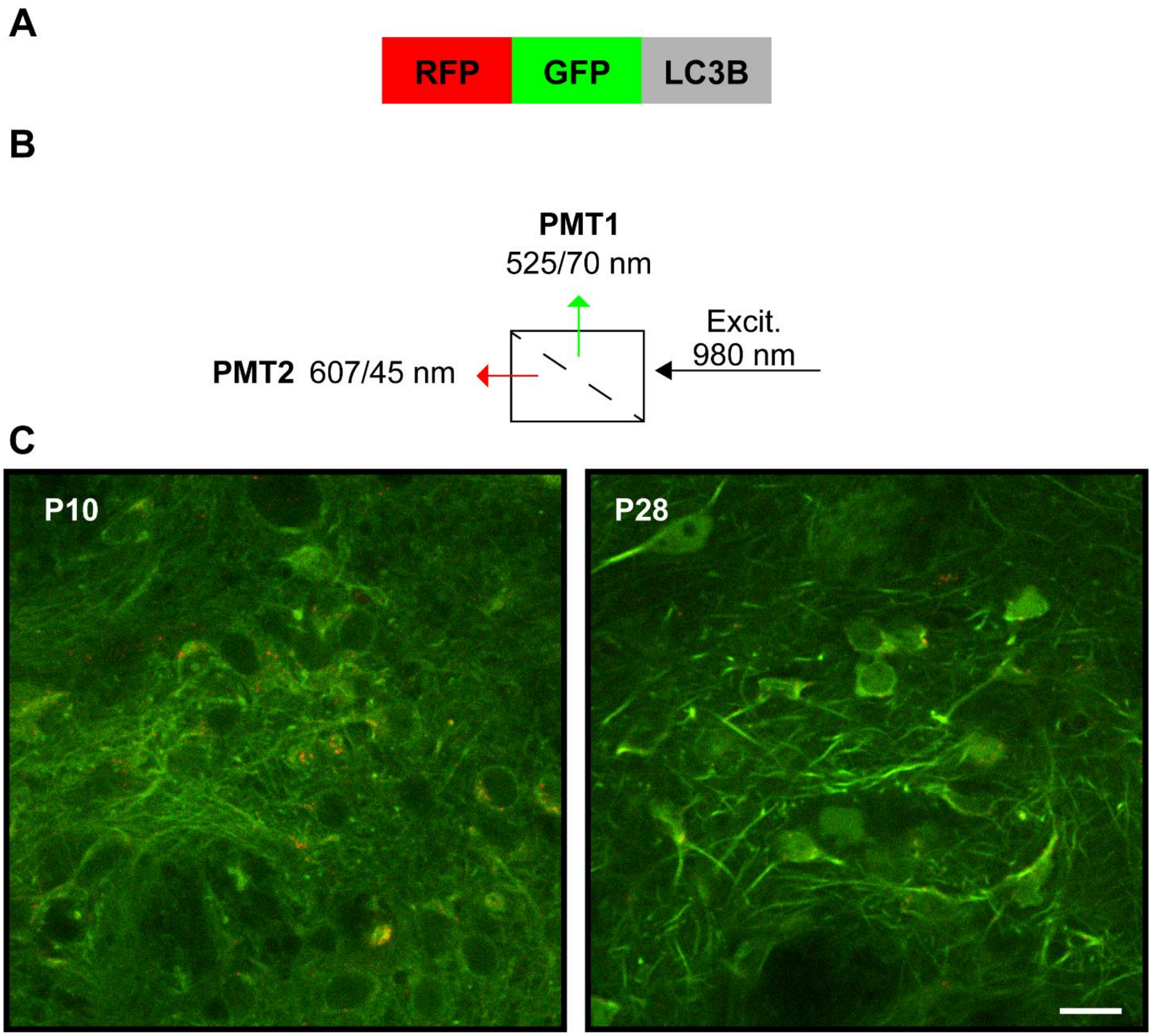

Figure S1. Live 2-photon imaging of tfLC3 in striatal slice. **(A)** Schematic representation of tfLC3 reporter. LC3B fused to RFP and GFP allows detection of cytosolic and membrane bound LC3 by fluorescence microscopy. GFP fluorescence is quenched in acidic environments, permitting analysis of autophagosome and autolysosome numbers. **(B)** Schematic of imaging setup. **(C)** Representative images of tfLC3 mice shows diffuse GFP imaging and punctate RFP patterns at both ages. Scale Bar 25 µm.
